# Supplementary figures and images for: Toll-1-dependent immune evasion induced by fungal infection leads to cell loss in the Drosophila brain
Source: PLoS Biol. 2025 Feb 13;23(2):e3003020. doi: 10.1371/journal.pbio.3003020 (PMC11825051; doi:10.1371/journal.pbio.3003020)

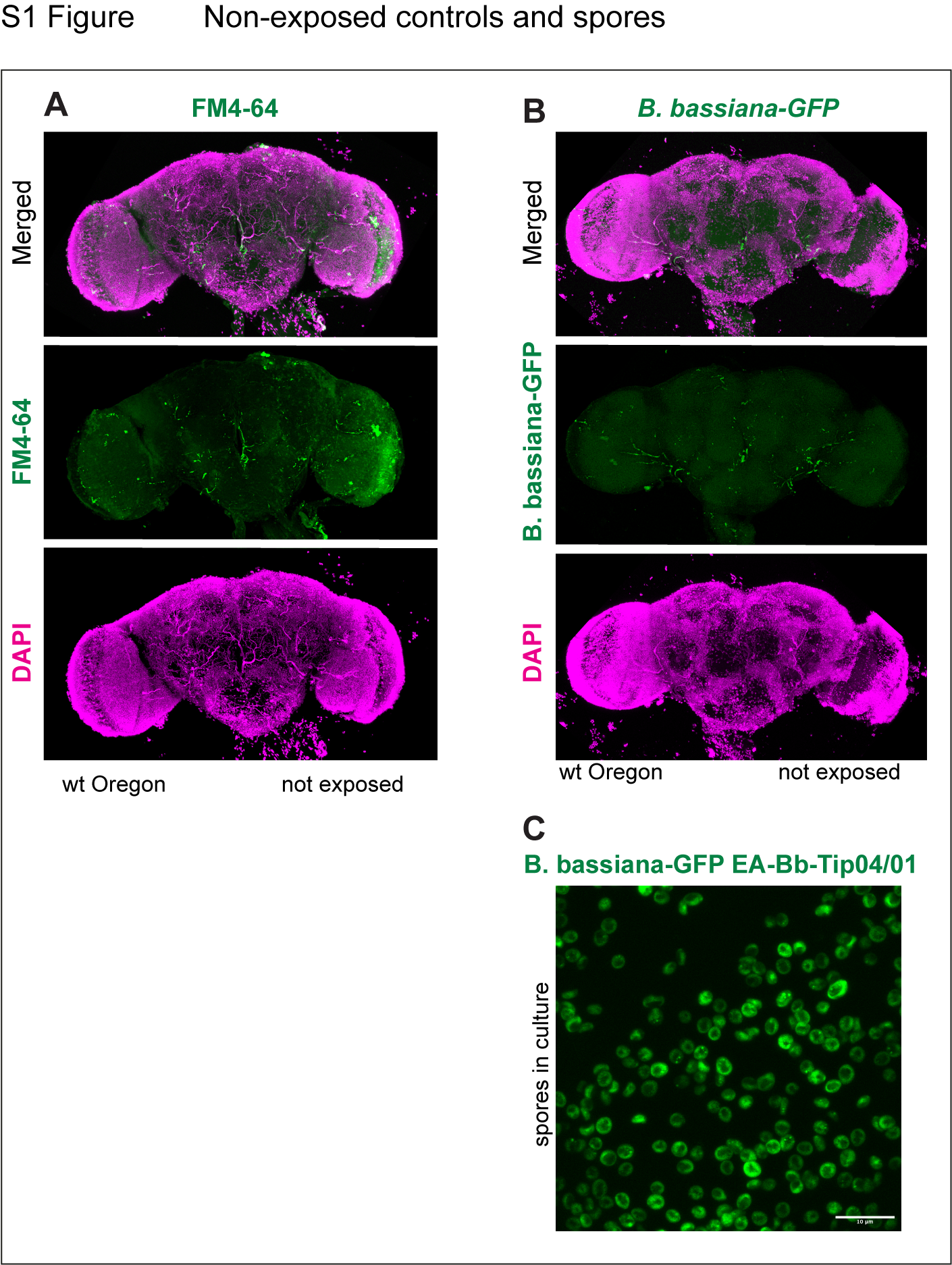

Supplement: S1 Fig — (A) Wild-type brains not exposed to fungi, treated and stained with FM4–64, at the same time and in the same way as flies exposed to B. bassiana in Fig 2A. Tracheae are often visible in confocal stainings of the brain producing no specific signal. (B) Wild-type brains not exposed to fungi, stained with anti-GFP, at the same time and in the same way as flies exposed to B. bassiana-GFP shown in Fig 2C-E. Tracheae are often visible in confocal stainings of the brain producing no specific signal and anti-GFP can give a background signal. (TIF) [file pbio.3003020.s001.tif]

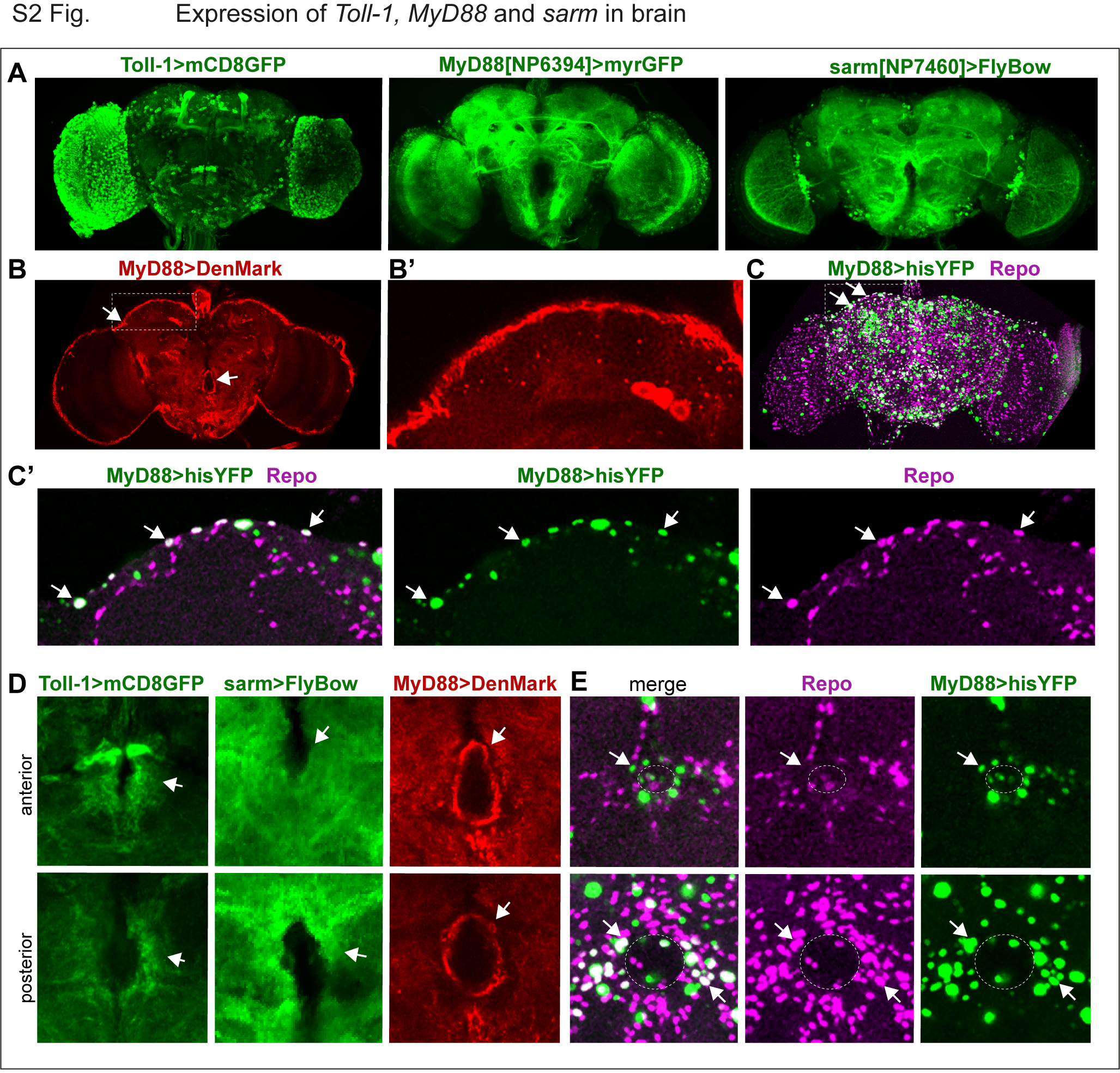

Supplement: S2 Fig — (A) Toll-1 > mCD8-GFP, MyD88NP6,394 > myrGFP and sarmNP7,460 > FlyBow reveal that these genes are expressed widely throughout the adult brain. (B) MyD88 > DenMark reveals the BBB surrounding the brain, detail in (B’). (C,C’) Colocalisation of nuclear markers YFP and the pan-glial nuclear marker anti-Repo (arrows) along the BBB, in MyD88 > hisYFP flies. The BBB is Drosophila if is formed of glial cells, and MyD88+ glia line up the outer edge of the brain (arrows), detail in (C’). (D) Detail from brains in (A,B) showing that Toll-1 > mCD8-GFP, MyD88NP6,394 > DenMark and sarmNP7,460 > FlyBow are localised at the entry point (anterior) of the proboscis into the brain and exit (posterior brain) of esophagous and neuropiles from the brain (arrows). (F) Colocalisation of YFP and Repo in glial cells of the BBB between proboscis and esophagus entry/exit through the brain (arrows) in MyD88 > hisYFP flies. (TIF) [file pbio.3003020.s002.tif]

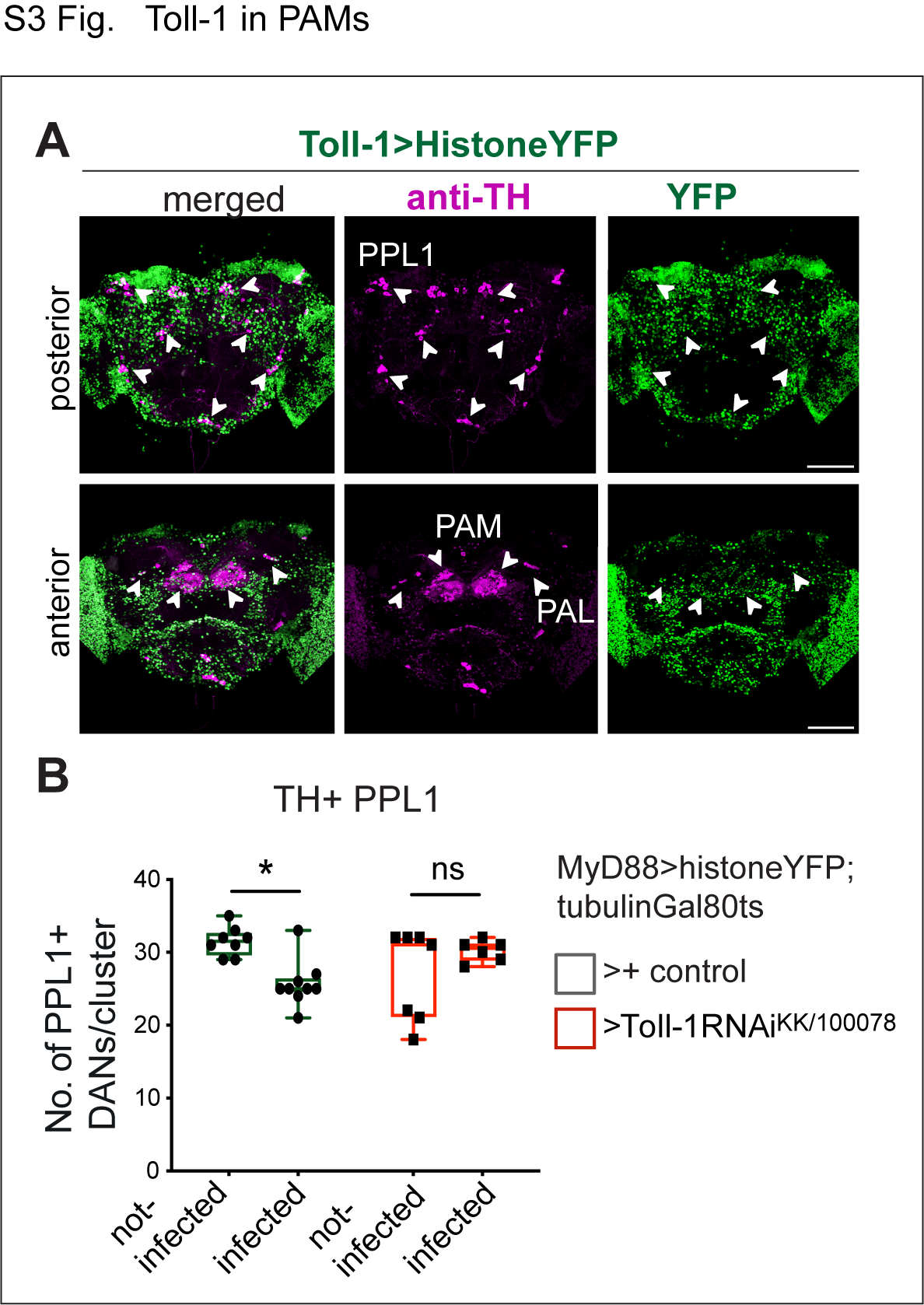

Supplement: S3 Fig — (A) Co-localisation of Toll-1 > histone-YFP with anti-TH in PPL1, PPL2, PPM3, PAL and a subset of PAM DANs, in adult fly brains. (B) Adult-restricted Toll-1-RNAi knock-down in MyD88 + cells does not affect PPL1 neurons, but it rescues the loss of PPL1s caused by B. bassiana infection. Two-way ANOVA: Infected versus not-infected p = 0.9586; Genotypes: p = 0.3628; Interaction: p = 0.0024, followed by Tukey’s multiple comparisons correction test. Sample size Toll-1 > histone-YFP fly brains n = 5, UAS-histone-YFP/+ , n = 5, non-infected control brains n = 8, infected control brains n = 9, non-infected Toll-1 KK/100078 RNAi brains n = 7, infected Toll-1 KK/100,078 RNAi brains n = 6. Graphs show box plots around the median. Asterisks on graphs: *p < 0.05, **p < 0.01, ***p < 0.001, ****p < 0.0001. The data underlying S3B Fig can be found in S5 Table Source data. (TIF) [file pbio.3003020.s003.tif]

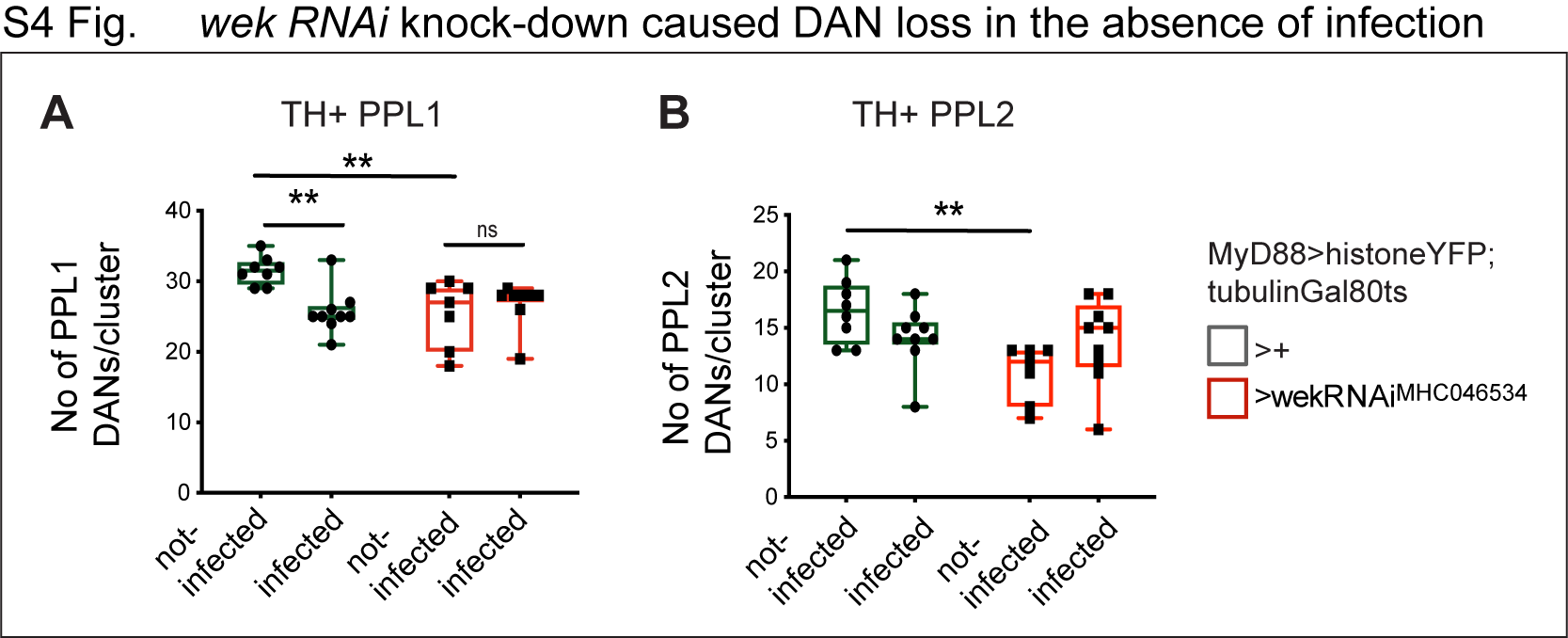

Supplement: S4 Fig — wek-RNAi knock-down caused a decrease in TH + DANs PPL1 (A) and PPL2 (B), that was not rescued with the over-expression of wek caused by B. bassiana infection, suggesting that wek is required for DAN differentiation. (A) Two-way ANOVA: Infected versus not-infected p = 0.0455; Genotypes: p = 0.0695; Interaction: p = 0.0038 followed by Tukey’s multiple comparisons correction test: non-infected control brains n = 8, infected control brains n = 9, non-infected wek-RNAi brains n = 7, infected wek-RNAi brains n = 9. (B) Two-way ANOVA: Infected versus not-infected p = 0.0104; Genotypes: p = 0.8564; Interaction: p = 0.0217 followed by Tukey’s multiple comparisons correction test: non-infected control brains n = 8, infected control brains n = 9, non-infected wek-RNAi brains n = 7, infected wek-RNAi brains n = 9. Graphs show box plots around the median. Asterisks on graphs: *p < 0.05, **p < 0.01, ***p < 0.001, ****p < 0.0001. The data underlying S4A and S4B Fig can be found in S5 Table Source data. (TIF) [file pbio.3003020.s004.tif]

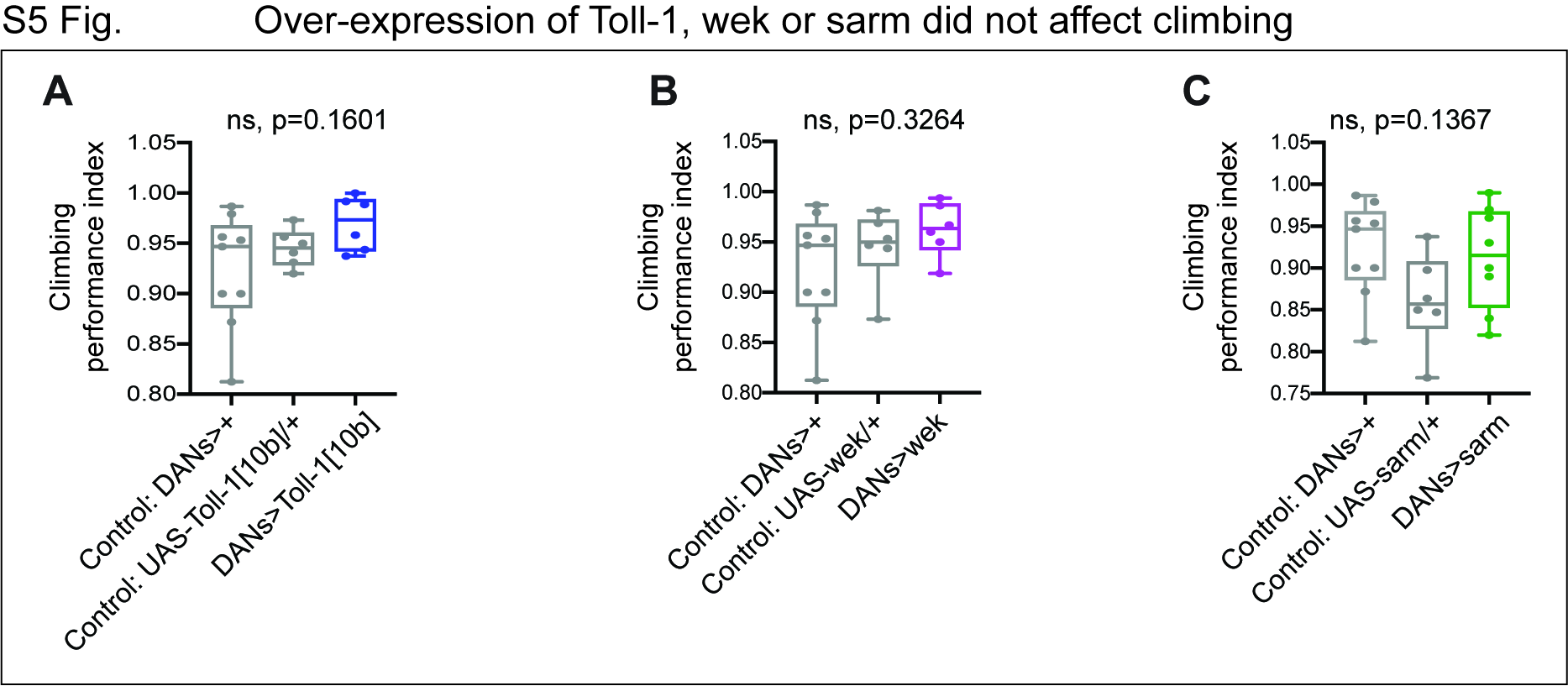

Supplement: S5 Fig — (A) Over-expression of activated Toll-110b in DANs. OneWay ANOVA: p = 01601, controls: THGAL4/+; R58E02GAL4/+. n = 76 flies; UAS-Toll-110b/+: n = 59 flies; GAL4/UAS-Toll-110b n = 82 flies. (B) Over-expression of wek in DANs. OneWay ANOVA: p = 0.3264. controls: THGAL4/+; R58E02GAL4/+. n = 76; UAS-wek-HA/+: n = 41 THGAL4/+; R58E02GAL4/UAS-wek-HA n = 59 flies. (C) Over-expression of sarm in DANs. OneWay ANOVA p = 0.1367. Controls: THGAL4/+; R58E02GAL4/+ . n = 76 flies; UAS-sarm/+: n = 61 flies; THGAL4/+; R58E02GAL4/UAS-dsarm n = 60 flies. Genotypes: (A) THGAL4/UAS-Toll-110b; R58E02GAL4/+; (B) THGAL4/+; R58E02GAL4/UAS-wek-HA; (C) THGAL4/+; R58E02GAL4/UAS-dsarm. Controls: THGAL4/+; R58E02GAL4/+ and UAS lines crossed to wild-type (Oregon). The data underlying S5A, S5B and S5C Fig can be found in S5 Table Source data. (TIF) [file pbio.3003020.s005.tif]
