# Supplementary material for: Toll-1-dependent immune evasion induced by fungal infection leads to cell loss in the Drosophila brain
Source: PLoS Biol. 2025 Feb 13;23(2):e3003020. doi: 10.1371/journal.pbio.3003020 (PMC11825051; doi:10.1371/journal.pbio.3003020)
Supplement: S1 Table — This includes all the Drosophila strains used in this work, their genotypes and origin. (DOCX) [file pbio.3003020.s007.docx]

**S1 Table Stock list**

| **S.No** | **Name** | **Full genotype** | **Source** |
| --- | --- | --- | --- |
| 1 | Sarm [NP7460] Gal4 | yw;P{w+ GawB} Ect4 NP7460/TM6B UAS lacZ | Kyoto #105471 |
| 2 | Sarm [NP0257] Gal4 | yw;P{ GawB} NP0257/TM6, P{UAS- lacZ.UW23-1} | Kyoto #103571 |
| 3 | MyD88 Gal4 histoneYFP; tubGal80ts | MyD88 [NP3694] Gal4 UAS histone YFP/CyO Gal80; tubGal80^ts^ | Hidalgo lab |
| 4 | UAS Flybow 1.1 | FB1.1[2609]/Gla Bc (line 4) | Gift from  Iris Salecker |
| 5 | UAS Histone YFP | w;UAS HistoneYFP;+ | Hidalgo lab |
| 6 | UAS myrGFP | W; UAS myrGFP | BSC |
| 7 | UAS myr-td-Tomato | w-; +; 10X UAS myr-Td-tomato | B. Pfeiffer |
| 8 | Toll-1 Gal4 | w;+/(CYO);Toll-1 Gal4 [CRISPR]/ TM6B | This work |
| 9 | UAS TrpA1 | w;UAS TrpA1 [attP216] | BDSC: 26263 |
| 10 | UAS Toll-1 RNAi | w;UASToll1RNAi(VDRC100078);+ | VDRC 100078 |
| 11 | UAS Wek RNAi | y sc v; UAS wek RNAi {TRIP MHC 046534} attp40 | BDSC: 57260 |
| 12 | UAS Sarm RNAi | y v; P{TRIP JF01681}attP2 | BDSC: 31175 |
| 13 | UAS Toll-1[10b] | w;UAS Toll-1[10bB] | Gift of Jean-Marc Reichhart |
| 14 | UAS Wek-HA | w;+/(CYO); UAS wek-HA[NA12]/ TM6B | Gift from Jean-Luc Imler |
| 15 | UAS-dsarm | w; UAS-dsarm | Gift of Marc Freeman |
| 16 | repoGAL4 | w; repoGAL4/TM6B | Hidalgo Lab |
