# Supplementary material for: Toll-1-dependent immune evasion induced by fungal infection leads to cell loss in the Drosophila brain
Source: PLoS Biol. 2025 Feb 13;23(2):e3003020. doi: 10.1371/journal.pbio.3003020 (PMC11825051; doi:10.1371/journal.pbio.3003020)
Supplement: S2 Table — The table includes the primer name, target gene and primer sequence. (DOCX) [file pbio.3003020.s008.docx]

**S2 Table List of primers**

| **Primer No.** | **Original name** | **Gene** | **Primer Sequence 5-->3** |
| --- | --- | --- | --- |
| 1 | GAPDH Fw qPCR | GAPDH | GTGAAGCTGATCTCTTGGTACGAC |
| 2 | GAPDH Rev qPCR | GAPDH | CCGCGCCCTAATCTTTAACTTTTAC |
| 3 | Sarm fw qPCR | Sarm | AATTCGGCGGAGATGAAACG |
| 4 | Sarm Rev qPCR | Sarm | GGCTTCCTTAATGACGGCTG |
| 5 | Wek Fw qPCR | Wek | AGAAGCCCTGTATATGCCCCG |
| 6 | Wek Rev qPCR | Wek | TGCTTGTTAAGAATCGCCCGTGT |
| 7 | Dros Fwd qPCR | Drosomycin | CCCTCTTCGCTGTCCTGATGC |
| 8 | Dros Rev qPCR | Drosomycin | GCACCAGCACTTCAGACTGG |
| 9 | Mtk Fwd qPCR | Metchnikowin | TTCTGGCCCTGCTGGGT |
| 11 | Mtk Rev qPCR | Metchnikowin | ACCCGGTCTTGGTTGGTTAGG |
| 12 | TH Fwd qPCR | TH | CGAGGACGAGATTTTGTTGGC |
| 13 | TH Rev qPCR | TH | TTGAGGCGGACCACCAAAG |
| 14 | 5’ HA Fwd | Toll-1 | ATGCGACCGGTAAAATCTCGTATTATGCAGCACTCGA |
| 15 | 5’ HA Rev | Toll-1 | GGAACTGAGCGGCCGCTGCAAATGGAGAAATTGAAAGGAAT |
| 16 | 3’ HA Fwd | Toll-1 | GATGGCGCGCCGTGAACCCATTTGGACAACA |
| 17 | 3’ HA Rev | Toll-1 | CGTACTAGTGCAGTTCAGCTCTCAGCCGT |
| 18 | Sense gRNA | Toll-1 | GTCGCCCATTTGGACAACATGAGTCGA |
| 19 | Antisense gRNA | Toll-1 | AAACTCGACTCATGTTGTCCAAATGGG |
