# Supplementary material for: Toll-1-dependent immune evasion induced by fungal infection leads to cell loss in the Drosophila brain
Source: PLoS Biol. 2025 Feb 13;23(2):e3003020. doi: 10.1371/journal.pbio.3003020 (PMC11825051; doi:10.1371/journal.pbio.3003020)
Supplement: S3 Table — It includes the names of the primary and secondary antibodies used for immunostaining experiments, the fluorophore linked to secondary antibodies, and the working dilutions. (DOCX) [file pbio.3003020.s009.docx]

**S3 Table List of antibodies**

| **Antibody** | **Donor** | **Working dilution** |
| --- | --- | --- |
| **Primary antibodies** | | |
| Anti-GFP | Rabbit | 1:250 |
| Anti-REPO | Mouse | 1:20 |
| Anti-ELAV | Rat | 1:250 |
| Anti-TH | Rabbit | 1:250 |
| **Secondary antibodies** | | |
| Anti-rabbit-488 | Donkey | 1:500 |
| Anti-mouse-546 | Goat | 1:500 |
| Anti-mouse-488 | Goat | 1:500 |
| Anti-rat-647 | Goat | 1:500 |
