# Supplementary material for: Toll-1-dependent immune evasion induced by fungal infection leads to cell loss in the Drosophila brain
Source: PLoS Biol. 2025 Feb 13;23(2):e3003020. doi: 10.1371/journal.pbio.3003020 (PMC11825051; doi:10.1371/journal.pbio.3003020)
Supplement: Attachment — Submitted filename: RESPONSE TO REVIEWERS PBIOLOGY-D-24-02233R1.pdf [file pbio.3003020.s014.pdf]

LETTER OF RESPONSE TO REVIEWERS

Singh et al *Fungi activate Toll-1 dependent immune evasion to induce cell loss in the host brain*

PLoS Biology MS: PBIOLOGY-D-24-02233R1

Reviewers comments shown in blue.
Page and line numbers refer to **TRACKED VERSION**, pages and line numbers in clean version can differ.

Reviewer #1 (Evidence, reproducibility and clarity (Required)):

Singh et al. report that, after exposure to the entomopathogenic fungus Beauveria bassiana, the Drosophila adults impaired fly locomotion and died within two weeks. During which time, the authors designed experiments and showed the decline of brain cells via a Toll-1/Wek/Sarm pathway, mimicking the neurodegenerative diseases in humans in association with fungal infections. Providing that the rather solid genetic evidence was shown for the pathway in mediating fly brain cell losses, critical issues of experiment design/setup and conclusion validity were concerned.

We would like to thank Reviewer 1 for taking the time to assess our work and for their constructive criticisms that helped improve it. We are very pleased that Reviewer 1 found the genetic evidence for the pathway mediating *B. bassiana* induced cell loss rather solid. Please allow us to respond to the concerns raised. Please also see our Revision Table, indicating the experiments we carried out to improve the work, at the end of this document.

Specific comments:
The fungus-exposed flies died within two weeks were largely typical. However, it was unclear how those flies could be uniformly contaminated with fungal spores in the infection chamber shown in Fig. 1A, by landing on fungal "carpet"? It is publicly known that entomopathogenic fungi (EPF) like B. bassiana infect insects via spore germination on cuticle and then penetration of cuticles by fungal hyphae/mycelia (e.g., Trends Microbiol. 2024. 32, 302-316).

Standard approaches to infecting *Drosophila* and mosquitos with fungi include spraying or dusting of insects with spores and a common approach in Toll signalling research is to infect Drosophila by pricking flies with an infected needle (Fehlbaum et al 1994 JBiol Chem; Lemaitre et al 1996 Cell; Gottar et al 2006 Cell; Cui et al 2019 Nat Comm). However, we wanted to avoid pricking, as it is an injury, and as our lab works also in injury responses in the CNS, we suspect this would compound the phenotypic response to the fungus. Thus, we aimed to recreate conditions that insects encounter upon natural exposure to fungi and were inspired by the field studies of Mailtand (1994, Proc Royal Soc Lond) and Elya et al (2018, eLife). Furthermore, Inamdar et al (2010) showed that exposure to fungal volatiles was sufficient to induce neurodegeneration, meaning fungal exposure could suffice. Matiland (1994) and Elya et al (2018) isolated fungi-infected flies from the wild. Subsequently, Elya et al (2018) established a method to expose Drosophila to *E. muscae,* whereby healthy flies were house together with infected cadavers in a confined space. In our set-up, a fungal culture was grown for 10 days in a glass bottle, and this fungal culture included hyphae and spores. Flies were inserted into the bottle and shaken, enabling flies to get directly exposed to spores. Flies in the bottles were then kept at 25°C for several days depending on the experiment (e.g. 1, 3, 7), after which only active flies were selected for further experiments. Infecting flies using this chamber produced consistent and robust results, as praised by all three Reviewers.

To clarify this, we have now added further details on the experimental set-up in the manuscript text, page 5 lines 135-137 and in Materials and Methods section, page 17, lines 719-726.

It is typical that EPF killed and mycosed insects within 5-14 days after topical infection by immersion in or spraying spore suspensions, or dusting on the sporulated plates. Fungal spores can be ingested by insects, largely those with chewing mouthparts. However, fungal spores can barely survive the highly-alkaline foreguts. It is questionable that flies could ingest spores and spores "infiltrated" the brains.

As explained above, our setup provides abundant exposure of flies to spores – similarly to immersion, spraying or dusting with spores - whilst mimicking natural conditions, and it yielded robust results.

Indeed, in principle, fungal spores would not survive in the foregut. Furthermore, gut microbes can reduce the virulence of fungi against insects (Peng Y et al., 2023, *J. Fungi 9*, 906). However, Ishii et al (2017, Scientific Reports) demonstrated that *B. bassiana* causes mosquito death only when ingested through the proboscis. **This is consistent with our feeding assay data, which demonstrate that adult flies did ingest spores (Fig 3A).**

In this context, it is relevant to note that the proboscis traverses the brain. This could provide *B. bassiana* with an infection route that avoids entering the gut any deeper. In fact, it has been previously proposed that fungi may colonise the brain to create a fungal reservoir that avoids the hostile environment of the gut (Elya et al 2018 eLife). Furthermore, Ishii et al (2017, Scientific Reports) demonstrated that following ingestion through the proboscis, *B. bassiana* invaded the brain.

The presence of fungi inside insect brains has been previously reported by other labs. Several entomopathogenic fungi have been demonstrated to enter the brains of their hosts, including Drosophila (Humber, 1976; Elya et al, 2018 eLife; Elya, 2023). Similarly*, B. bassiana* was detected in the brains of mosquitos following ingestion (Ishii et al 2017, Scientific Reports). Even in humans, fungal spores have been found in brains of Parkinson's Disease patients (Chen et al 2022 J Fungi; Alonso et al 2014 J Alzheimer's Dis; Cannon and Gruenheid, 2022 TIM). These published reports use multiple methods, but they do not always qualify whether they were visualising spores, hyphae or mycelia. **Consistent with these findings, our data also show the presence of fungi in the brain.**

The evidence we had provided that *B.bassiana* penetrates the brain are that Using FM464 (new Figure 2A), calcofluor white (new Figure 2B) and *B. bassiana-GFP* (new Figure 2C-F) we could detect colocalization of the three fungal markers with the nuclear marker DAPI inside the brain. We also detected germlings in the brains of *B. bassiana-GFP* infected flies (new Figure 2F', F'').

In response to this Reviewer's feedback, we now provide further new data:

- **New Figure 2C,D,E:** in flies exposed to *B. bassiana-GFP ,* we show *B. bassiana-GFP* within the brain (Figure 2C,D, at the location of entry of the proboscis into the brain (Figure 2C,E) and crossing the blood brain barrier (BBB)(Figure 2D).

- **New Figure 2F-F'' and New Figure 3B'':** additional, higher magnification images of germinating spores (also called germlings) in the brains of exposed flies (Figure 2F'') and un-exposed flies fed with B.bassiana-GFP (Figure 3B'').

- **New Figure 3B-B'''':** un-exposed flies were fed water containing *B.bassiana-GFP* spores and their brains were dissected. This revealed *B.bassiana-GFP* cells within the proboscis (Figure 3B'), at the entry point of the proboscis into the brain (Figure 3B''') and at the exit of the oesophagus from the brain (Figure 3B'''), and over glial cells within the brain (Figure 3B''''). Germlings were also found in the brain.

- Previous reports did not qualify whether spores or hyphae were detected (Elya et al 2018 eLife; Ishii et al 2017 Scientific Reports). Consistently with this, **we have now edited the term "spores" for a generic "fungal cells" for the results using FM4-64 and calcofluor white, see page 5 line 155, 159.** We have kept the term spores, germlings or germinating spores for the results obtained with *B. bassiana-GFP*, e.g. **page 6 line 202-209.**

- We have added text on these points in: **page 6 lines 202-209 and Discussion page 12 line 501 to Page 14 line 601.**

Regarding the detection of fungal cells in fly brains, on the one hand, the authors argued that detection of fungal SPORES in fly brain THREE days post exposure (page 5) by infiltration. It would be impossible that, even fungus could breach the blood brain barrier (BBB), it might be the fungal hyphae/mycelia but not the spores. One the other hand, the authors provided the evidence of the damaged BBB SEVEN days post exposure, a few days LATER than the detection of fungal spores in brains (THREE days) post treatment mentioned above. Did "spore infiltration" (even impossible) occur before BBB damage?

1. **<u>On 'impossible':</u>** the presence of fungal bodies, fungal cells, fungal spores, hyphae or mycelia within the brain had already been demonstrated by multiple labs (see comments above and below). Some previous reports did not qualify whether spores or hyphae were detected (Elya et al 2018 eLife; Ishii et al 2017 Scientific Reports). Consistently with this, and in response to this Reviewer, **we have now edited the term "spores" for "fungal cells" for the results using FM4-64 and calcofluor white.** We have kept the term spores, germlings or germinating spores for the results obtained with *B. bassiana-GFP*. In the original version, we demonstrated the presence of GFP labelled transgenic *B. bassiana-GFP* germlings, germ tubes or germinating spores inside the *Drosophila* brain (original Figure 1G). Please compare the extremely similar shape of our germlings with those described by Lewis et al (2009 Microbiology).

Several entomopathogenic fungi can enter the brains of their hosts, including *Drosophila* (Humber, 1976; Elya et al 2018; Elya et al 2023; Ishii etl al 2017). For example, Elya et al (2018, eLife) demonstrated the infiltration of *E. muscae* into the Drosophila brain; Ishii and colleagues demonstrated the presence of *B. bassiana* inside the brain of mosquitos (Ishii et al 2017 Scientific Reports). Fungal spores have also been found inside the brains of human Parkinson's disease patients (Chen et al 2022 J Fungi; Alonso et al 2014 J Alzheimer's Dis; Cannon and Gruenheid, 2022 TIM). **In our work, compelling evidence that *B. bassiana* penetrates the brain had been provided with three fungal markers, most particularly with a GFP-tagged strain of *B. bassiana* (**strain EA-Bb-Tip04/01 **new Figure 2C-F).**

<u>In response to this Reviewer's feedback, we have expanded the evidence and now provide new data that *B.bassiana* entered the brain:</u>

- **Figure 3F,F': *B. bassiana-GFP* co-localising with the nuclear marker DAPI, indicative of spores.**

- **Figure 3F,F'': *B. bassiana-GFP* germlings with clear filaments, following fly exposure.**

- **Figure 3F,F'': *B. bassiana-GFP* germlings in non-exposed flies, after feeding on *B.bassiana-GFP*.** Here, flies were not exposed to the fungus in an infection chamber. Instead, they were kept in an empty cage and were fed on *B.bassiana-GFP* spores mixed into water. Thus, flies only acquired *B.bassiana-GFP* through feeding. After feeding, *B.bassiana-GFP***+** cells and germlings were found within the dissected brain. Shown in **new Figure 3F,F''.**

We had provided further, indirect, evidence that B. bassiana entered the brain with the activation of Toll signalling within the brain upon infection. Infection with *B. bassiana* upregulated the expression of Toll signalling target anti-microbial genes, including *dros* and *mtk*, and Toll signalling adaptors, *wek* and *sarm*, as

revealed by **qRT-PCR in dissected and carefully cleaned up brains (Figure 3F,G).** Activation of Toll signalling requires the cleavage of its ligand Spz by a protease cascade, which starts by the detection of the pathogen by the pattern recognition receptor GNBP3. *GNBP3* is expressed in the brain, and so is *spz,* its protease *SPE*, and *Toll-1*. Fungal volatiles are not known to activate Toll signalling. Furthermore, our data are consistent with those of Elya and colleagues (2018 eLife) who used RNAseq of *E. muscae* infected brains to show that genes responsible for innate immunity, including also the Toll signalling adaptor *wek*, were upregulated upon infection. **Hence, *B. bassiana* induced activation of Toll signalling within the brain means that fungi were present within the brain.**

2. <u>**On the BBB:**</u> We visualised BBB breakdown at 7 days because this was the time-point we had focused on for the functional analysis, but breakdown is likely to have started earlier. In fact, infection of *Drosophila melanogaster* with *E. muscae* induced blood-brain barrier (BBB) permeability, which became more severe over time (Elya, 2023 eLife). RNAseq analysis in *E. muscae* infected brains revealed the up-regulation of Toll-dependent signalling at 24-48h post-exposure (and was sustained until 96h), fungal cells were detected within the brain at 48h post-exposure and BBB permeability was most pronounced after 69h post-exposure (Elya et al 2018 eLife). This means that Toll signalling was the first sign of the infection reaching the brain, followed by detection of fungal cells within the brain, and lastly BBB breakdown. Most likely, this does not mean that this is the sequence in which events took place, but rather reflects detection thresholds, with qRT-PCR/RNAseq being the most sensitive method. **Our findings are entirely consistent with these findings.**

The exact mechanism by which BBB permeability increased with infection was not identified by Elya et al (2018, 2023). In ***Drosophila*, the BBB is formed by glia cells, and our work showed that exposure to *B. bassiana* caused glial cell loss (original Figure 3C,D, new Figure 4C,D). This is consistent with exposure to *B. bassiana* causing damage to the BBB, as we had shown in original Figure 1H (new Figure 2G).**

<u>**In response to this Reviewer's feedback, we have now provided clarifications and new data:**</u>

- **Added a clarification:** "Importantly, loss of glial cells could lead to BBB breakdown and facilitate entry of *B. bassiana* into the brain". **page 8 line 317-318.**

- **New Figure 2C,D,E showing that *B.bassiana-GFP* crosses the BBB.** The BBB is Drosophila is formed of glial cells. The outer most layer of the brain is formed of surface glial cells, and their nuclei can be seen with DAPI **(Figure 2C,D)** and the glial marker Repo **(New Supplementary Figure 2C,C').** In **(Figure 2C,D)** *B.bassiana-GFP+* cells can be clearly seen traversing the BBB surrounding the central brain at multiple points. They can also be seen traversing the BBB at the point of entry of the proboscis into the brain **(new Figure 2E).**

- **New Supplementary Figure 2A,B,C,D,E: showing that *MyD88* is expressed in glial cells of the BBB.** We had shown that *B.bassiana* infection caused BBB breakdown (Figure 2G), glial cells loss (Figure 4C,D), loss of MyD88+ cells (Figure 5A,B), and that infection-dependent loss of both MyD88+ and Repo+ cells requires Toll-1 (Figure 5A-D). Thus, expression of MyD88 in glial cells of the BBB means that *B.bassiana* could damage the BBB by inducing glial cell death and thus holes in the BBB. A comment has been added to **page 9 lines 370-372.**

- **New Figure 2E showing *B.bassiana-GFP* cells at the cell boundary as the proboscis penetrates the anterior brain and also as the oesophagus exits the posterior brain, in exposed flies**. It is important to note that the proboscis – which the fly uses for feeding – penetrates the brain, there merges into the oesophagus, which exits the brain to join the gut. As they traverse the brain, they form a tunel delimited by a BBB formed of glial cells. Here, flies exposed to *B.bassiana-GFP* in an infection chamber had GFP+ cells throughout the brain, including at the entry and exit points of the proboscis/oesophagus.

- **New Figure 3B''' showing *B.bassiana-GFP* cells at the entry and exit points of the proboscis/oesophagus through the brain, in non-exposed flies that were fed on *B.bassiana-GFP.***

- **New Supplementary Figure 2A,B,C,D,E showing that the tunnel that traverses the brain for the proboscis/oesophagus is ensheathed in a BBB formed of Repo+ glial cells that express MyD88.** Moreover, this BBB expresses MyD88 (Supplementary Figure 2D,E), as the BBB can be clearly seen (MyD88>Denmark, Supplementary Figure 2D) and the MyD88-YFP+ cells also express the glial marker Repo (Supplementary Figure 2E)

- **New Supplementary Figure 2D showing that as the proboscis/oesophagus traverse the brain, they are surrounded by the expression of *Toll-1, MyD88* and *sarm.*** This means that *B.bassiana-GFP* will be able to activate Toll-1 signalling as soon as it penetrates the BBB.

Altogether, these data show that *B.bassiana* penetrates the BBB, Toll signalling is present at the BBB, and B.bassiana induces glial cell loss. This means that *B.bassiana* can damage the BBB via Toll-1-dependent cell loss.

The authors stated that "by day seven more than half of the flies had died" (Fig. 1B). It is questionable therefore that the "other half" of the diseased and dying insects were used for the following experiments. There would be no wonder that the climbing of these diseased and dying flies was impaired, however, which could be due to muscle damage, hemocyte number decline and reduction of energy production etc. apart from brain cell loss. The brain function of dying animals could be compromised by multiple direct or indirect factors.

The survival curve in Figure 1B showed that at 7 days around 40% of flies were alive. In our set-up, a fungal culture was grown for 10 days in a glass bottle, and this fungal culture included hyphae and spores. Two-day-old flies were inserted into the bottle and shaken, enabling flies to get directly exposed to spores. Flies in the bottles were then kept at 25°C for several days, after which only active flies were selected for experiments. Flies that were immobile, dying or stuck to the fungal culture were not used. To prepare flies for the climbing experiments, selected flies were inserted into fresh vials and flipped 2-4 times, to remove excess spores from their cuticles. The flies were then placed in the climbing assay vials and after 30 mins of habituation the climbing experiments were performed. Infecting flies using this chamber produced **consistent and robust results, as praised by all three Reviewers.**

Indeed, climbing could be affected by multiple reasons. Presumably fungal volatiles alone are sufficient to induce climbing impairment, as has been shown before (Inamdar et al 2013 PNAS). They showed that the climbing impairment caused by exposure to octanol correlated with the loss of dopaminergic neurons (Inamdar et al 2013 PNAS). Loss of dopaminergic neurons causes locomotion impairments in flies, as well as humans (Inamdar et al 2013 PNAS; Sun et al 2018 Frontiers Syst Neurosci). **Our work showed that, as well as this mechanism, fungi can also directly activate Toll signalling to induce dopaminergic cell loss in the brain**.

In response to this Reviewer's feedback, we have now:

- **A clarification has been added in page 19 lines 778-784.**

- **New Supplementary Video 1 (Oregon x Cantons_C4_S10_7do):** we also add a video – data from Figure 1B - showing that flies that were exposed to *B.bassiana* for seven days were moving and could climb, although not as well as un-infected controls.

Issue of Fig. 2D labelling.

Done.

Referee Cross-Commenting

I agree with that Reviewers 2 and 3 that rather solid evidence of fly brain loss was shown in this work, however, at most in association with exposure to fungal cultures (volatiles could not be excluded etc.). "Spores" entry into fly brains were suspicious or impossible. If the dying flies had been used for these neurological experiments, the reliability of conclusions would be highly concerned.

We are very pleased that all three Reviewers agree that our evidence of cell loss after exposure to *B.bassiana* is solid. Regarding the other points, please see our detailed response above. In brief:

(1) We provide new data evidence of GFP+ germlings within the brain after exposure or ingestion of *B.bassiana-GFP*. Germlings have been used by others as evidence of germinating spores within the brain.

(2) We provide new data evidence that *B.bassiana-GFP* can enter the brain through the proboscis after ingestion.

(3) We provide video evidence that infected flies were not dying as they could move, just not as well as un-infected flies.

Please see above our detailed response to these points.

Reviewer #1 (Significance (Required)):

Since there are critical concerns of experiment designs/setup in this work, it is questionable that fly brain cell loss was caused by fungal entry into brains.

We would like to thank Reviewer 1 for taking the time to assess our work and for their constructive feedback that helped improve it. In response, we have now provided abundant new, higher resolution data that show that *B.bassiana* can enter the brain.

****

Reviewer #2 (Evidence, reproducibility and clarity (Required)):

Summary

The authors describe a role for Toll signaling in detrimental neuronal loss associated with B. bassiana fungal infection in Drosophila melanogaster model. They show that this effect is mediated by wek/sarm as silencing either of them prevents neuronal loss after the infection. Similar results are obtained with Toll-1 RNAi, suggesting that the response is dependent on the activation of Toll signaling by B. bassiana. The study is well executed, main conclusions are backed up by the data presented and experiments are conducted with adequate numbers of replications and individuals. Below I give some comments that I think would help in further improving the manuscript.

We would like to thank Reviewer 2 for the positive evaluation of our work and for the criticisms that helped improve it further. Please allow us to respond to the points raised. Please also see our Revision Table, with the experiments we have carried out and the new data we now provide that improved the manuscript.

Major comments

As the initial experiments (including the effect on survival and climbing assay) have been performed using OR/CantonS, it would be interesting to investigate if the same is seen with a more similar background to that what is used in the genetic experiments. In addition, I'd suggest an experiment to see if the Toll (or wek/sarm) RNAi in the brain rescues the climbing defect caused by the fungal infection.

We have done these experiments. **We first tested whether all the controls could climb normally and they did (see figure below).** This showed that the GAL4 drivers themselves would be unlikely to participate in the phenotypes. Since the cell biology was carried out mostly with MyD88GAL4, we carried out the knock-down experiments using this line, to link to the cell biology data.

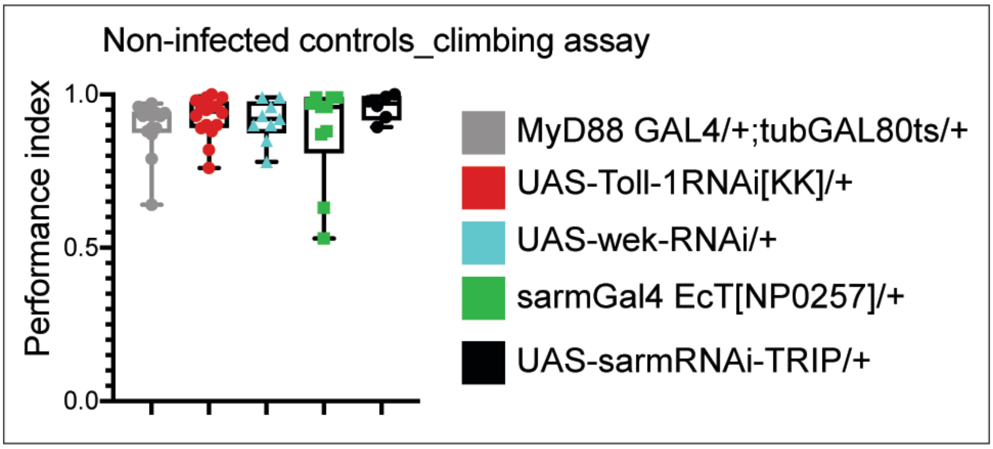

To address these two points raised by this Reviewer, we have carried out new tests using MyD88GAL4 and provide new data:

- **New Figure 5I, Figure6I, Figure 7I survival:** shows that the **MyD88/+; tubGAL80^{ts}/+** (ie F1 outcross of MyD88GAL4/CyO; tubGAL80^{ts}/tubGAL80^{ts} to wild-type Oregon) **control genotype** has no impact on survival. In fact, 100% un-infected flies of this genotype survive after 7 days. Furthermore, exposure to *B.bassiana* has a similar effect in fly survival in this genotype as in wild-type (Oregon/CantonS F1 in Figure 1B), with a median 30% surviving flies after 7 days of exposure to *B.bassiana.*

- **New Figure 5J, Figure6J, Figure 7J climbing:** shows that the ***MyD88/+; tubGAL80^{ts}/+* control genotype** has no overall impact on climbing either. In fact, non-infected flies of this genotype achieve a climbing Performance Index around 1, comparable to that of Oregon/CantonS wild-type flies. Furthermore, exposure to *B.bassiana* has a similar effect in climbing in this genotype as it does in wild-type (Oregon/CantonS F1 in Figure 1B), with a statistically significant difference in climbing in infected vs not-infected flies after 7 days of exposure ($p<0.0001$ for both genotypes). Climbing is slightly more severely impaired in infected *MyD88/+; tubGAL80^{ts}/+* flies than in infected wild-type flies. This can be explained if the GAL4 insert into the *MyD88* locus affects the function of MyD88 (which is unknown), which could weaken flies specifically upon infection.

- **New Figure 5I,J, Figure 6I,J and Figure 7I,J survival and climbing:** we also provide **new controls,** the F1 progeny of the **UAS-RNAi lines to wild-type Oregon (i.e. UAS-Toll-1RNAi/+, UAS-wekRNAi/+ and sarm-RNAi/+).** The results are again consistent, as in un-infected flies survival and climbing are unaffected. After 7 days of exposure to *B.bassiana,* these genotypes show a consistent decrease in fly survival and a decrease in climbing performance.

- **New Figure 5I,J, Figure 6I,J and Figure 7I,J show the effect of knocking-down *Toll-1, wek* or *sarm* in survival and climbing, after 7 days of exposure to *B.bassiana.*** We show that *Toll-1* knock-down improves slightly survival after 7 days of exposure to B.bassiana. Remarkably, *Toll-1* knock-down rescues the climbing impairment caused by B.bassiana infection **(New Figure 5J)**. This is consistent with the observed Toll-1-dependent cell loss upon infection. Interestingly, wek knock-down did not have an effect, consistently with the pleiotropic functions and compound phenotypes of interfering

with its function **(New Figure 6I,J)**. Importantly, knocking down sarm improved both survival and the climbing ability of flies **(New Figure 7I,J).** The phenotypes of knocking-down *Toll-1* were more pronounced than those of knocking down *wek* or *sarm.* This suggests that anti-microbial peptides may also induce neurodegeneration via Toll-1, as shown for the IMD pathway. However, esolving this point was beyond the scope of this revision.

- **New Supplementary Figure 5A,B** shows that although the **over-expression of *Toll-1, wek* or *sarm*** caused loss of some PAM neurons, this was not sufficient to impair clim   bing in the absence of infection. This can be explained as only a fraction of PAMs was lost in each genotype, and more neurons than just PAMs control climbing.

Altogether, our new data show that knock-down of *Toll-1* (Two Way ANOVA genotype factor: $p<0.05$) and *sarm* (Two Way ANOVA gentotype factor: $p<0.05$) rescues the climbing deficit caused by exposure to *B.bassiana* (see statistical details in Supplementary Table S4), consistently with the cell loss data.

It is somewhat unclear what are the controls in the genetic experiments. For example, in Figure 2, the control is UAS-TrpA1/+. Does this mean that the UAS-TrpA1 flies have been crossed to something (like the driver background strain) or used as it is? In Figure 4, controls are ">+". Again, are MyD88>histoneYFP;tubulinGal80ts flies crossed to something (in this case, maybe the w1118 background of the KK library RNAi strains) or used as homozygous? And same for the subsequent figures. I'd ask the authors to clarify these points in the manuscript.

Thank you for this feedback. The controls were F1 progeny flies resulting from crossing GAL4 driver lines or UAS responder lines to a wild-type stock (Oregon). UAS-TrpA1/+ stands for F1 progeny flies of the cross: UAS-TrpA1 x wild-type (oregon). The symbol “>” means GAL4/UAS, and this is common practice in the field. In Figures 4-7, “>+” stands for F1 progeny flies of the cross of a GAL4 driver line to wild-type (Oregon). The line MyD88GAL4/CyO; tubGAL80^{ts}/tubGAL80^{ts} was crossed to wild-type Oregon and F1 progeny flies MyD88GAL4/+; tubGAL80ts/+ were used as controls. To make this genotype more readable in figures, it was abbreviated as tubGAL80ts, MyD88>+ , and this is also common practice.

This has now been clarified in Materials and Methods, **page 16 lines 697-704. And a stock list is provided in Table S1.**

We have also now given all the genotypes in the figure legends and we also provide an **excel file that contains all statistical analysis details and includes all the genotypes.**

Could the authors please explain why they opted for MyD88-GAL4 in the experiments in Figures 5-7?

To target the innate immunity pathway. The innate immune response goes via Toll-1, MyD88, Dif/NFκB and AMP upregulation. Wek is known to facilitate the interaction between Toll-1 and MyD88 at the plasma membrane (Chen et al 2006 Curr Biol). However, Wek is not involved in innate immunity (Chen et al 2006) and plays multiple other pleiotropic roles instead (Li et al 2020 eLife). One of these functions is to bring Sarm close to the Toll signalling complex at the membrane (Foldi et al 2017 JCB). Sarm cannot bind Tolls directly, but it can bind Wek, which binds to Tolls and MyD88 (Foldi et al 2017 JCB). Sarm is the highly evolutionarily conserved and universal inhibitor of MyD88 (Carty et al 2006 Nat Immunol; Foldi et al 2017 JCB). However, Sarm is also well known to carry out other functions independently of Toll-1, e.g. NADAse and axon destruction (Summers et al 2016 PNAS). MyD88 is expressed in both neurons and glia (Li et al 2020 eLife). The blood brain barrier (BBB) is formed of glia, and B. bassiana damages the BBB. Thus, by using MyD88 GAL4 we aimed to target cells that respond to a fungal infection via Toll-1, that affect MyD88+ cells and that also involve Sarm as the main inhibitor of MyD88 signalling, ie leading to immune evasion.

We have expanded this explanation in **page 8, lines 314-318 and 331-335.**

What is the overall expression pattern of MyD88-GAL4?

We published the pattern of MyD88GAL4 in the adult brain before, it is widely expressed in the adult brian, in subsets of both neurons and glia (Li et al 2020 eLife). But we wanted to address this question more in depth, in the context of this work.

Thus, in response to this point, we have carried out new experiments and provide new data in:

- **New Supplementary Figure 2A:** showing the expression in the adult brain of *MyD88, Toll-1* and *sarm.* These data show that they are all widely expressed across the adult brain.

- **New Supplementary Figure 2A,B,C,D,E: showing that *MyD88* is expressed in glial cells of the BBB.** We had shown that *B.bassiana* infection caused BBB breakdown (Figure 2G), glial cells loss (Figure 4C,D), loss of MyD88+ cells (Figure 5A,B), and that infection-dependent loss of both MyD88+ and Repo+ cells requires *Toll-1* (Figure 5A-D). Thus, expression of MyD88 in glial cells of the BBB means that *B.bassiana* could damage the BBB by inducing glial cell death and thus holes in the BBB. A comment has been added to **page 9 lines 370-372.**

- **New Supplementary Figure 2E showing that the proboscis/oesophagus tunnel that traverses the brain is ensheathed by a BBB formed of Repo+ glial cells that express *MyD88.*** Moreover, that this BBB expresses *MyD88* can be clearly seen with MyD88>Denmark **(new Supplementary Figure 2D).**

- **New Supplementary Figure 2D shows that as the proboscis/oesophagus traverse the brain, they are surrounded by the expression of *Toll-1, MyD88* and *sarm.*** This means that *B.bassiana-GFP* will be able to activate Toll-1 signalling within the brain as soon as it crosses or damages the BBB.

Is there a possibility that some of the effects seen could arise from the Toll/sarm/wek knockdown elsewhere in the fly?

Toll/wek/sarm RNAi knock-down was used to rescue cell loss in the brain caused by exposure to *B. bassiana,* using three distinct markers: MyD88-HIsYFP+ (GAL4-reporter), Repo+ (with antibodies) and TH+ (antibodies) cells. We did not carry out tests to drive knock-down elsewhere and not in the brain, and test the effect in the brain. This would have been a large task, well beyond a revision for this work. Thus, we cannot rule out a possibility that knocking-down Toll-1 e.g. in the gut would not compound the brain phenotype. However, **we have now shown that Toll-1, MyD88 and sarm are widely expressed in the adult brain (new Supplementary Figure 2A)**, and the **experiments were well controlled** (as praised by the three Reviewers).

Furthermore, **we also show now that *Toll-1* and to some extent *sarm* knockdown can rescue the climbing impairment caused by infection (new Figure 5J and new Figure 7J) – and climbing behaviour is a readout of neuronal function.** Thus, altogether, it is extremely unlikely that the rescues within the brain resulted simply from RNAi knock-down outside the brain, in other organs or tissues.

How do the flies survive the infection with Toll knockdown in MyD88-epressing cells (expressed at least in all immunogenic tissues)? A bit more explanation would clarify the situation.

Our exposure to *B.bassiana* is different from the pricking method commonly used in Toll research. A common approach in Toll signalling research is to infect Drosophila by pricking flies with an infected needle (Fehlbaum et al 1994 JBiol Chem; Lemaitre et al 1996 Cell; Gottar et al 2006 Cell; Cui et al 2019 Nat Comm). Upon pricking with an infected needle, Toll-1 mutant flies die after 2 days. Please note that those mutant

flies were mutant throughout development, ie a cellular context in which the whole organism lacked immunity for the entire duration of their existence.

However, we wanted to avoid pricking, as it is an injury, and as our lab works also in injury responses in the CNS, we were concerned that this would compound the phenotypic response to the fungus in the brain. Thus, with our exposure chamber we aimed to recreate conditions that insects encounter upon natural exposure to fungi and were inspired by the field studies of Mailtand (1994, Proc Royal Soc Lond) and Elya et al (2018, eLife). We also wanted **to knock-down *Toll, wek* and *sarm* signalling specifically and only in adult flies and** in the context of exposure to *B.bassiana,* to separate the response from other functions (e.g. developmental, brain). For example, we have shown that Tolls also have other functions in the brain (Li et al 2020 eLife).

Thus we: (1) did not use pricking but exposure; (2) we did not use mutants, but instead we knocked-down Toll-1 in adult flies, after eclosion – that is, immunity had been normal until then. (3) RNAi produces a hypomorphic knock-down, but not a loss of gene expression like with a null allele. Altogether, our manipulation is considerably milder and more restricted than using Toll mutants.

We were also surprised to find that knock-down of *Toll-1* in MyD88 cells improved fly survival after 7 days of exposure to *B.bassiana* and improved climbing. We had previously shown that all the RNAi lines used work well (Li et al 2020 eLife; Foldi et al 2017 J Cell Biol). Perhaps AMPs also induces neuro-degeneration, and we showed that exposure to *B.bassiana* increased AMP levels, and these would be regulated by Toll-1 but not Sarm. In fact, others have shown that antimicrobial peptides downstream of Toll signalling can also induce neurodegeneration (Dubei and Tapadia, 2018 Molec Neurobiol; Shukla et al 2019 Cell Rep; Baek et al 2024 Mol Brain). This would explain why the rescue of survival and climbing from knocking down *Toll-1* is greater than for knocking down *sarm*.

We have now added a comment in the Discussion session, **page 15 lines 651-652.**

Minor comments
Page 3: Full species name should be given here (Drosophila melanogaster)
Done.

A short description of the FM4-64 dye (what it stains etc) would be useful for the readers unfamiliar with it.
We have now added this to the Materials and Methods section**, page 21 lines 855-859.**

FM4-64 is a red fluorescent dye (excitation/emission maxima ~515/640 nm) used to stain yeast vacuolar membranes (Roeder and Shaw, 1996 Genetics). FM4-64 dye has been reported to stain B. bassiana spores in-vivo in the haemolymph of tobacco budworm (Lewis et al., 2009 Microbiology), suggesting its potential use in staining spores in the adult Drosophila brain. Calcofluor white is another fluorescent dye which binds to 1-3 beta and 1-4 beta polysaccharides of chitin and cellulose present in fungal cell walls (Hoch et al., 2005).

Page 6: Please explain shortly why TrpA1 overexpression was used to activate the neurons.
In brief, it is a standard method to activate neurons. TrpA1 is a cation channel involved in heat perception. It is closed at lower temperatures, and it opens at 30°C, letting Na+ and Ca++ into the neuron, triggering an action potential. In transgenic flies, this makes it possible to activate any neurons of choice, under the control of a promoter-GAL4, by moving flies from 18 to 30°C for some time. It is commonly used in *Drosophila* neuroscience.

We have added an explanation in Materials and Methods section, **page 19 lines 801-803.**

Figure 2E: What is the genotype of the flies? mtk is lacking statistics
The genotype is wild-type (Oregon), and this information has now been added.

Page 8: third row refers to Figure 2 but should be Figure 3.
Corrected.

Although antibody stainings are performed using "standard methods", a short overview on the process should be presented also in the current manuscript. Also, I imagine fungal spores are all over the flies retrieved from the infection chamber. I'd like to know (and this could also be described in the materials) how the flies (and the brains) were treated/washed prior to preparing brains for immunostaining and imaging?

We have now added further details in the Materials and Methods section**, page 23 lines 921 to page 24 935.**

To prepare flies for immunostaining, **i**nfected flies were first transferred to vials from infection chamber and flipped twice to remove excess spores. The flies were then transferred to 70% ethanol and washed in 1XPBS for 10 minutes. Adult Brains were dissected in 20 minutes and fixed in 650ul fixative (650ul 4% paraformaldehyde (PFA) in 1X Phosphate buffer saline (PBS) at room temperature for 20 minutes in a 1.5ml Eppendorf. The dissected adult brains were thoroughly cleaned removing all fat body, other surrounding cells and tissues. Subsequently, the brains were washed for 20 mins in 750ul of 0.5% PBT (47.5ml 1xPBS + 2.5ml 10% Triton) 5 times.

Some typos and inconsistencies at various places. For example, at some occasions B. bassiana written without a space in between "B" and "bassiana" and not in italics (both in figures and in text); on page 5, first line: "mimicked" misspelled
Corrected.

Referee Cross-Commenting

As the fungal infiltration into the brain is central to the conclusions made in the manuscript, I agree that care should be taken in making this argument solid. I believe this can be achieved adding controls as reviewer #3 suggests together with additional experiment(s) verifying that Toll/wek/sarm in the brain is mediating the neuronal loss caused by the fungal infection (rescue experiments). Of note, I wonder if, similarly to mammalian macrophages, hemocytes could be responsible for delivering the fungal cells into the brain?

Please see also our response to Reviewer 1 above. Summarised here briefly, **on *B. bassiana* entering the brain:**

- **We provide new evidence that *B.bassiana-GFP* enters the brain following exposure** in the infection chamber **(new Figure 2C,D,E,F)**

- **We provide new evidence that *B.bassiana-GFP* enters the brain after feeding** non-exposed flies with *B.bassiana-GFP* **(new Figure 3B-B''").**

- We show in both cases ***B.bassiana-GFP* traversing the brain**: both **at the entry point of the proboscis into the brain and exit of the oesophagus in exposed flies (new Figure 2E)** and **in *B.bassiana-GFP* fed flie**s **(new Figure 3B''');**

- we show that following **feeding un-exposed flies with *B.bassiana-GFP*, GFP+ cells are found within the proboscis and all the route into the brain (new Figure 3B-B''');**

- we provide new images of **germlings or germinating fungal spores within the brain**, both in exposed flies **(new Figure 2F', F'')** and in flies fed with ***B.bassiana-GFP*** **(new Figure 3B'')**

- we provide new evidence that *B.bassiana-GFP* **crosses the BBB (new Figure 2C,D),**

- that ***MyD88*** **is expressed in cells of the BBB,** surrounding the central brain and the tunnel through which proboscis and oesophagus traverse the brain **(new Supplementary Figure 2B,B', C,C', D, E)**

- and that ***Toll-1, MyD88*** **and** ***sarm*** **are all expressed in cells surrounding the tunnel** across the brain through which the proboscis and oesophagus traverse it **(new Supplementary Figure 2D),** meaning *B.bassiana* can activate this pathway as soon as it enters the brain via this route.

We had already shown in the original submission that exposure to *B. bassiana* causes glial cell loss. The BBB in *Drosophila* is formed of glial cells. Our data show that *B. bassiana* can enter the brain through the BBB surrounding the brain including also the BBB between proboscis and brain. As infection causes glial cell loss, then most likely *B. bassiana* damages the BBB in this way, making its way into the brain.

On the suggestions by Reviewer 2, we have now provided:

- Non-infected controls **(new Supplementary Figure 1)**

- Tested whether RNAi knock-down of *Toll-1/wek/sarm* can rescue the climbing impairment caused by exposure to B. bassiana: **new Figure 5I,J, Figure 6I,J and Figure 7I,J.** Remarkably, *Toll-1* RNAi knockdown significantly rescues the climbing impairment caused by exposure to *B.bassiana. sarmRNAi* knockdown also showed this trend, although the effect was milder (significant in the Two Way ANOVA test genotype comparison p=0.0290, but not in post-hoc tests). This can be due to other factors contributing to the neurodegeneration caused by *B.bassiana:* Inamdar et al (2013 PNAS) have shown that exposure to fungal volatiles is sufficient to cause neurodegeneration and impair climbing and AMPs can also induce neurodegeneration (Dubei and Tapadia, 2018 Molec Neurobiol; Shukla et al 2019 Cell Rep; Baek et al 2024 Mol Brain).

There is evidence that macrophages can cross the BBB in Drosophila (Winkler et al 2021 Sci Adv). Thus, it is possible. However, investigating this is a large task, beyond the scope of this revision. Furthermore, as the BBB in Drosophila is made up of glial cells, and we showed that exposure to *B. bassiana* causes glial cell loss (ie Repo+ cells, Figure 4C,D), and we now show that MyD88 is expressed in glial cells of the BBB **(new Supplementary Figure 2B-E)** then *Toll-1*/MyD88 dependent loss of glial cells at the BBB is likely responsible for causing BBB damage. **These data and explanation are given in the revised version of the manuscript.**

I agree with the reviewer #1 that the climbing defect could be because of multiple reasons other than the fungal spores in the brain causing neuronal loss (for instance flies being generally weak at this point,). However, the authors do show convincingly that there is neuronal loss in fungal-infected flies.

Indeed, we cannot rule out that climbing deficits could be due to an extended infection throughout the body, or to the exposure to fungal volatiles. Inamdar et al (2013 PNAS) showed that fungal volatiles impair climbing in *Drosophila.* Also, others have shown that AMPs downstream of both Toll-1 and IMD pathways can induce neurodegeneration in *Drosophila* (Dubei and Tapadia, 2018 Molec Neurobiol; Shukla et al 2019 Cell Rep; Baek et al 2024 Mol Brain). In fact, these factors explain why knock-down of *Toll-1, wek* and *sarm* do not lead to a robust rescue of survival or climbing, which we tested for this revision. This does not diminish the validity of our findings, but instead reveals the complexity of host-pathogen interactions with relevant implications for health.

Reviewer #2 (Significance (Required)):

Fungal infections are understudied in any research model considering the threat they pose to humans and other animals alike. Due to the high conservation of the signaling components studied here, the results provide a good basis for future research, extending to mammalian models. I think these results will be of interest to a wider audience because of the reasons stated above.

Thank you for appreciating the relevance of our findings.

My fields of expertise are Drosophila melanogaster, innate immunity, cell-mediated immunity, blood cell homeostasis

We would like to thank Reviewer 2 for the positive evaluation of our work and for the feedback that helped improve it further.

Reviewer #3 (Evidence, reproducibility and clarity (Required)):

In this manuscript, Singh et al. demonstrate that infection of D. melanogaster flies with B. bassiana fungus induces neurodegeneration via Toll/wek/sarm signalling. It is already known that fungal infection can be associated with neurodegeneration, but the exact mechanism is unclear. The authors demonstrate that the fungus enters the brain, causes hallmark symptoms of neurodegeneration, and requires Toll-1, Wek, and Sarm in order to do so. This is an important step forward as it demonstrates specific genes in the fly immune pathways that are involved in fungus-induced neurodegeneration, which could be informative for infections in humans. Overall, the manuscript is thorough and well-written and the conclusions are broadly supported. A few mostly minor comments and questions are below, which could mostly be addressed by including additional details in the methods or discussion.

We would like to thank Reviewer 3 for the positive evaluation of our work, and for the feedback that helps improve it further. Please allow us to respond to each of the points raised below. Please also see our Revision Table, indicating the experiments we carried out to improve the work.

The only major comments would be

1) that the control fly genotypes used in experiments were not always the most ideal controls (eg, compared WT genotypes to RNAi against a gene of interest; ideally would be RNAi against a control gene compared to RNAi against a gene of interest), and

Reviewer 3 is correct in pointing out that this would have been a good control for RNAi. However, it is not common practice, as most publications using RNAi do not carry this out. Unfortunately, we are not able to add this at this stage, as to use controls appropriately, it would mean repeating everything, which is beyond the scope of this revision. Nevertheless, our data are on the whole robust against the controls used, as praised by all three Reviewers.

2) negative controls of fluorescence microscopy imaging were not always included. It would be important to address these through clarification in the figures/methods, and/or discussion of the potential caveats, even though it is likely the conclusions would still hold. Notably, these comments are relatively easily addressed through edits in the text.

Unfortunately, we did not keep non-infected controls when carrying out the calcofluor white stainings, but we did for the rest. Thus, we **have now provided:**

- **new Supplementary Figure 1A:** non-infected controls stained with FM4-64
- **new Supplementary Figure 1B:** Non-infect control brains stained with anti-GFP to compare with *B. bassiana-GFP.*

In both sample types, the trachea comes up non-specifically, which is extremely common in brain stainings and microscopy. However, the qualities of the signal in test samples differ (see Figure 2).

Major comments:
• For fluorescence imaging, were negative controls included (no infection or no gene expression etc.) for all stains (as with Figure 1H)? If so, it would help to include representative images as supplemental figures.
As explained above, we **have now provided the controls for FM4-64 and *B. bassiana-GFP*, in a new Supplementary Figure 1A,B.**

Also, for all positive samples, was presence of the fungus noted in all samples?
We have now provided this information in the **figure legend, revised Figure 2** and we also provide a **Supplementary Table S4 with sample sizes, genotypes and statistical details.**

• Figure 4: Here, it appears that the control fly genotypes are wildtype vs an RNAi line (similar for some other figures/assays as well, but using this one as an example). The best control would be RNAi against a control gene compared to RNAi against a gene of interest, rather than just a control WT genotype with no RNAi compared to RNAi against a gene of interest.

This should be included as a caveat in the discussion since the experiments do not all account for the effect of RNAi (or other gene expression) on the phenotypes regardless of the gene.

Reviewer 3 is correct in pointing out that this would have been a good control for RNAi. However, it is not common practice, as most publications using RNAi do not carry this out. Unfortunately, we are not able to add this at this stage, as to use controls appropriately, it would mean repeating everything, which is beyond the scope of this revision. Nevertheless, our data were on the whole robust against the controls used, as praised by all three Reviewers.

We have now discussed this in page 14 lines 616-619.

Minor comments:
• It would help to have line numbers throughout
We have now added line numbers to manuscript.

• Figure 1- what are the arrows in panels D-G?
Fungal cells in the brain, visualised with different fungal markers, and scanned under the confocal microscope for two different channels. The images are shown as merged image containing both channels, and as split channels, to see each raw data more clearly. The arrows indicate the position of the fungal cells that colocalise with the nuclear dye DAPI (Figure 2A,F), or next to the neuronal nuclear marker Elav (Figure 2B), indicating this same position in the merged channel and in the split channels.

We have now added the arrows into the figure legends, as they were missing. Thank you for drawing this to our attention.

• Methods: A few details are unclear:
o Was only one fly sex used or were both used for the various assays? If both were used, were they statistically assessed for differences? Sex is only mentioned in a couple of the methods sections.

We used both females and males for experiments in Figures 1-4, but we used exactly the same number of each gender for all experiments. We found no differences between sexes. For this reason, for all other experiments from Figure 5 onwards we used only females, as they are bigger, so their brains are also bigger and easier to dissect.

We have now included this information in the Methods section, page 24 lines 936-939.

o How old were the flies at the start of the experiments? A few experiments noted age, but it was not clear for all
Two-day-old flies were inserted into the chamber containing *B. bassiana*, and then kept at 25°C for several days, depending on the experiment. For experiments involving crosses, F1 progeny flies were collected for up to 2 days, and then placed in the infection chamber. Most experiments were carried out at 3 days or 7 days post-exposure, and this is explained in the text.

We have now included this information in the Methods section, **page 18 line 734-735.**

o For longevity, was the fungal culture ever replaced during the experiment?
Fungal cultures were replaced every 10 to 15 days. **We have now added this information in page 18, lines 737=738.**

o For the climbing assay when the flies were initially flipped, how much time was there between flips?
If we understand correctly, Reviewer 2 is asking about the flips prior to the climbing assay. The flies were first transferred from the infection chamber to the standard vials containing fly-food, and they were instantly flipped 2-3 times to eliminate excess spores from their cuticles, with 20-30 sec between flips. Subsequently, flies were transferred to the empty climbing assay vials. For the climbing assay itself, flies were filmed for 10 seconds, followed by 30'' rest, and this was repeated 15 times for each cohort.

Some of these details were already there, but a clarification has been added in page 19 line 782.

• Figures 2A, 2D, 2E, 3E, & 3H: If multiple replicates or samples are represented in the data, it would help to be able to see the data points underlying these bars. If so, please add them to the graphs to see the spread of data points.
Done.

• Figure 3F- what do arrows indicate?
The positions of the different classes of dopaminergic neurons, whose names are given on the side of each image (e.g. PPL1, PPM3, PPL2, PPM1/2). **We have now clarified this in the figure legend, page 39 lines 1685-1686.**

• It is interesting that Wek-RNAi with infection not only rescues loss caused by the infection alone, but also increases YFP cells beyond the uninfected controls (Figure 5C). The same is true with toll-1 RNAi (Figure 4C). Why might this be?
Both *Toll-1* and *wek* have been shown to induce apoptosis (Foldi et al 2017 JCB). Thus, it is possible that knocking them down enables cells that would normally die, to survive, compounding the phenotype.

We have added a comment in Discussion, page 14 line 620-622.

• It would be ideal if data underlying data points and full statistical models and outputs could be included through a public repository such as Dryad. This would be ideal for full assessment of statistical approaches

Please allow us to point out that most publications do not include all the statistical analysis data. Nevertheless, we now include a **Supplementary Table S4 that contains all the statistical details.**

Very Minor comments:
• Check italicizing throughout- missed a few "Drosophila" or "B. bassiana" in main text or figures
Done.

• Looks like no space between C. and elegans in C. elegans in a few cases
Done.

• Word missing: "No effect was seen after three days exposure to B. bassiana, but seven days exposure impaired climbing"... seven days of exposure?
Done.

• Toll-1 misspelled pg 6 last paragraph
Thank you, we have now corrected this.

Referee Cross-Commenting

Regarding the major comments, I agree with Reviewer 1 that more thorough proof of spores entering the brain (and what proportion of exposed flies this happens to) would be beneficial.

Please see our response to Reviewer 1. In summary:

- **We provide new evidence that *B.bassiana-GFP* enters the brain following exposure** in the infection chamber **(new Figure 2C,D,E,F)**

- **We provide new evidence that *B.bassiana-GFP* enters the brain after feeding** non-exposed flies with *B.bassiana-GFP* **(new Figure 3B-B'''').**

- We show in both cases ***B.bassiana-GFP* traversing the brain**: both **at the entry point of the proboscis into the brain and exit of the oesophagus in exposed flies (new Figure 2E)** and **in *B.bassiana-GFP* fed flie**s **(new Figure 3B''');**

- we show that following **feeding un-exposed flies with *B.bassiana-GFP*, GFP+ cells are found within the proboscis and all the route into the brain (new Figure 3B-B''');**

- we provide new images of **germlings or germinating fungal spores within the brain**, both in exposed flies **(new Figure 2F', F'')** and in flies fed with ***B.bassiana-GFP* (new Figure 3B'')**

- we provide new evidence that *B.bassiana-GFP* **crosses the BBB (new Figure 2C,D),**

- that ***MyD88* is expressed in cells of the BBB,** surrounding the central brain and the tunnel through which proboscis and oesophagus traverse the brain **(new Supplementary Figure 2B,B', C,C', D, E)**

- and that ***Toll-1, MyD88* and *sarm* are all expressed in cells surrounding the tunnel** across the brain through which the proboscis and oesophagus traverse it **(new Supplementary Figure 2D),** meaning *B.bassiana* can activate this pathway as soon as it enters the brain via this route.

We had already shown in the original submission that exposure to *B. bassiana* causes glial cell loss. The BBB in *Drosophila* is formed of glial cells. Our data show that *B. bassiana* can enter the brain through the BBB

surrounding the brain including also the BBB between proboscis and brain. As infection causes glial cell loss, then most likely *B. bassiana* damages the BBB in this way, making its way into the brain.

I also agree with Reviewer 2 that a rescue experiment for the climbing assay and my earlier suggestion for more controls in the microscopy could help address this concern, at least in part.

- We have now tested survival and climbing in flies where *Toll-1, wek* or *sarm* were knocked-down in MyD88+ cells (**new Figure 5I,J, new Figure 6I,J and new Figure 7I,J,** respectively), and also where they were over-expressed in dopaminergic neurons **(Supplementary Figure 5A-C).** These data show that Toll-1 knock down can rescued the climbing impairment caused by the infection.

- We provide a new Figure with the not-exposed controls that we had for FM4-64 and B. bassiana-GFP. Unfortunately, we did not keep them for calcofluor white at the time, and it would not be meaningful to repeat this one now as controls need to be carried at the same time. All other figures already have these controls. **See new Supplementary Figure 1: non-exposed controls.**

Other responses or experiments may also be appropriate to address some of the major concerns- maybe additional assay(s) of brain function other than climbing?

Thank you for the suggestion. However, we consider this beyond the scope of this manuscript. Behaviour experiments are not trivial. This work already took us more than 4 years of very hard work and it is not clear that another behavioural test would add information that the current data have not already provided.

Reviewer 1 also brought up the point that flies with advanced infection were used for the experiments- it would be helpful to know if earlier time points were ever checked for BBB damage, loss of brain cells, or presence of fungus etc. This would clarify if the same phenotypes are present in flies that die early, along with other concerns from Reviewer 1.

As explained above, after seven days of exposure to the fungal spores, only active flies were selected for experiments. Flies that were stuck to the fungal culture were not used. **We now include a video of climbing wild-type infected and non-infected flies, showing that all flies were mobile (new Supplementary Video S1).**

The presence of fungus within the brain was observed at 1-3 days of exposure to *B. bassiana.*

At the early stages of the project, we tested cell number after 3 and 7 days of exposure to *B. bassiana.* After three days of exposure to *B. bassiana,* we did not detect cell loss in the brain using *anti-Repo* or *sarm>hisYFP.* This means that it takes some time for *B. bassiana* to cause widespread neurodegeneration.

Please see our response to Reviewer 1 (above) on other points raised.

However, whether directly or indirectly, several later figures show loss of brain cells with infection followed by rescue with RNAi against genes of interest. This does lend support to the conclusions that fungal infection negatively impacts brain cells and the fungus requires these host genes to do so.

We are pleased that Reviewer 3 finds that our data support our interpretation and conclusions. Infact, we used three distinct methods to monitor cell rescue: (1) GAL4/UAS based His-YFP reporter, and cells were counted automatically and objectively using DeadEasy software. (2) Anti-Repo antibodies for all glia, and automatic DeadEasy cell counting; (3) anti-TH antibodies for dopaminergic neurons and Fiji-assisted manual cell counting.

Other concerns Reviewer 2 and I raised about the fly genetic controls being unclear should also be addressed. What is the full genotype of the flies in each case? What is considered "+" in each case? Were these driver background strains, WT (like Oregon R), or RNAi against control genes (best controls)?

Please see our response to Reviewer 2 above.

We have **now added explanations in the Materials and Methods page 17, lines 643-648**, added full genotypes in figure legends and added **a Supplementary Table S4** with statistical details, sample sizes and genotypes.

Reviewer #3 (Significance (Required)):

General assessment: The manuscript by Singh et al. is a thorough investigation into the fungus-host interactions in the brain, demonstrating that the common insect fungal pathogen B. bassiana requires the host genes Toll, wek, and sarm to induce negative phenotypes in the brain. The strengths are in the multi-pronged approaches that use several independent techniques (fly behavior assays, gene expression, microscopy, etc.) and multiple genes, conducted with many replicates, that all show clear and consistent trends supporting the conclusions of the authors. The weaknesses include some cases where controls are either not completely clear or not the most ideal controls. This weakness could be addressed with either edits to the text, where appropriate, or addition of supplemental figures. However, the conclusions are still broadly supported.

We are pleased that Reviewer 3 found that the data showed clear and consistent trends supporting our conclusions.

On controls, in response to Reviewers, we have:

- added a mention of the **RNAi-control caveat in page 14, lines 616-617.**
- provided **non-exposed controls for staining of B. bassiana, in new Supplementary Figure 1.**
- **tested whether the GAL4 driver line controls could affect climbing and they don't:** sarmGAL shown in the Letter of Response to Reviewers, response to Reviewer 2 (see above), **page 7 of this letter,** and MyD88GAL4 shown in **new Figures 5I,J, new Figure 6I,J and new Figure 7I,J.**
- added in the text further explanations of the **controls used and the symbolism** used to abbreviate genotypes **(e.g. page 17 lines 700-702)**

Advance: Although it is known that fungal infections can impair brain function, it is not fully understood how this happens. This manuscript identifies Toll-associated molecules that are required for fungus-mediated neurodegeneration, which is a critical first step to understanding the process and for future development of therapies.

Audience: This finding would be of broad interest to scientists in immunology, microbiology, neuroscience, and other areas.

We would like to thank Reviewer 3 for the positive evaluation of our work and helpful feedback that helped improve it.

Expertise of reviewer: Drosophila, fly genetics, invertebrate immunology, insect-fungal interactions

We thank all three Reviewers for their helpful feedback that helped improve our work.

Please find below a **Revision Table**, which lists the **experiments** we carried out for this revision and where the new data provided can be found . **For changes to the text,** see the Letter of Response to Reviewers and the submitted word **manuscript file with tracked changes.**

**LIST OF EXPERIMENTS DONE FOR THIS REVISION**			
**REVIEWER**	**CRITICISM**	**EXPERIMENT**	**NEW DATA**
**Reviewer 1**	1...... fungal spores can barely survive the highly-alkaline foreguts. It is questionable that flies could ingest spores and spores "infiltrated" the brains.	**Images showing B. bassiana-GFP at the point of entry of the proboscis into the brain, in flies exposed to *B. bassiana-GFP* in infection chambers.**	**New Figure 2C, D, E**
		**Un-exposed flies fed with *B. bassiana-GFP* showed GFP inside the proboscis and brain.**	**New Figure 3B’ proboscis<br>New Figure 3B’’’ and B’’’’ in brain**
		**Un-exposed flies fed with *B. bassiana-GFP* showed GFP at the entry and exit sites as proboscis traverses brain**	**New Figure 2E<br>New Figure 3B’’’**
	2. detection of fungal SPORES in fly brain THREE days post exposure (page 5) by infiltration. It would be impossible that, even fungus could breach the blood brain barrier (BBB), it might be the fungal hyphae/mycelia but not the spores.	**We have** now added new and higher magnification **images** of *B. bassiana-GFP* in brain, crossing the BBB	**New Figure 2C, D** shows orthogonal views through one single optical section (1 μm step)
		**We have** now added new and higher magnification **images** of *B. bassiana-GFP* germlings in the brain, after exposure and after feeding.	**New Figure 2F’’**<br><br>**New Figure 3B’’**
	3. ... authors provided the evidence of the damaged BBB SEVEN days post exposure, a few days LATER than the detection of fungal spores in brains (THREE days) post treatment mentioned above. Did "spore infiltration" occur before BBB damage?	New data showing B. bassiana-GFP crosses the BBB	**New Figure 2C, D** shows orthogonal views through one single optical section (1 μm step)
		New data showing that **MyD88 is expressed at the BBB** surrounding the brain and also the tunnel through which the proboscis/. oesophagus traverse the brain	**New Supplementary Figure 2B,B’,C,C’,D,E**
		New data showing that **Toll-1, MyD88 and sarm are all expressed in cells surrounding the entry/exit point of proboscis through the brain,** meaning *B. bassiana* entering the brain through proboscis can easily activate the pathway.	**New Supplementary Figure 2A,D**
	by day seven more than half of the flies had died" (Fig. 1B). It is questionable therefore that the "other half" of the diseased and dying insects were used for the following experiments..... The brain function of dying animals could be	Only flies that were alive and mobile following exposure to *B. bassiana* in the infection chambers were used for the experiments. We provide one example, in the uploaded video.	Supplementary Video 1

	compromised by multiple direct or indirect factors.		
**Reviewer 2**	1. As the initial experiments (including the effect on survival and climbing assay) have been performed using OR/CantonS, it would be interesting to investigate if the same is seen with a more similar background to that what is used in the genetic experiments.	**CLIMBING ASSAY AND SURVIVAL**<br><br>We tested all controls, ie MyD88GAL4 and sarmGAL4 drivers and all UAS lines and they all climbed well.<br><br>Thus, we used the MyD88GAL4 for the experiments in point (2), which include the controls	All controls including **sarmGAL4** given in page 7 of the letter of Response to Reviewers.<br><br>**MyD88 GAL4 controls given in:**<br><br>**New Figure 5I,J**<br>**New Figure 6I,J**<br>**New Figure 7I,J**
	2. To see if the Toll (or wek/sarm) RNAi in the brain rescues the climbing defect caused by the fungal infection.	**CLIMBING ASSAY AND SURVIVAL**<br><br>**Test rescue of infection-induced climbing impairment and survival by knocking-down Toll/wek/sarm in the brain**<br><br>Test in infected vs not-infected flies, with *B. bassiana,* 7 days in infection chamber:<br><br>**MyD88GAL4; tubGAL80ts x**<br>UAS-Toll-1RNAi[KK]<br>UAS-wekRNAi<br>UAS-sarmRNAi<br><br><u>Controls:</u><br>MyD88GAL4; tubGAL80ts x Oregon<br>UAS-Toll-1RNAi[KK] x oregon<br>UAS-wekRNAi x oregon<br>UAS-sarmRNAi x oregon	These new data are provided in:<br><br>**New Figure 5I,J**<br>**New Figure 6I,J**<br>**New Figure 7I,J**
		<u>**Additionally we tested:**</u><br>whether over-expression of *Toll-1[10b], wek* or *sarm* impair climbing in the absence of infection:<br><br>**THGAL4; R58E02GAL4 x**<br>**UAS-Toll-1[10b]**<br>**UAS-wek**<br>**UAS-dsarm**	**New Supplementary Figure 5**
	What is the overall expression of MyD88-GAL4?	We now provide data showing the expression of Toll-1GAL4, MyD88GAL4 and sarmGAL4 in the brain.	**New Supplementary Figure2A,D**
	Microscopy images of staining *B. bassiana* for non-infected controls	Provided for FM6-64 and *B. bassiana-GFP*	**new Supplementary Figure 1**

**Reviewer 3**	Reviewer 3 did not request any new experiments, but instead changes to the text.<br>(See tracked version of MS for changes to text).	**On controls:**<br>Microscopy images of staining *B. bassiana* for non-infected controls. Provided for FM6-64 and *B. bassiana-GFP*	**new Supplementary Figure 1**

For changes to the text, see the Letter of Response to Reviewers and the **submitted word manuscript file with tracked changes.**
