# Supplementary material for: Toll-1-dependent immune evasion induced by fungal infection leads to cell loss in the Drosophila brain
Source: PLoS Biol. 2025 Feb 13;23(2):e3003020. doi: 10.1371/journal.pbio.3003020 (PMC11825051; doi:10.1371/journal.pbio.3003020)
Supplement: Attachment — Submitted filename: LETTER OF RESPONSE TO REVIEWERS R2.docx [file pbio.3003020.s015.docx]

LETTER OF RESPONSE TO REVIEWERS – R2

Reviewer comments are in blue.

Reviewer #1

The authors performed additional experiments and carefully answered the questions raised by this reviewer. Beauveria cells entering into fly brain has been solidified and overall quality has been considerably increased in this revision.

This reviewer would like to keep the concern that the impaired locomotion of flies might not be solely due to the B. bassiana-induced neurodegeneration.

We have added new sentences explaining this point in page 5 lines 124-125 and page 14 lines 394-397 of tracked version.

Minor comments:
Figure 1C, statistic test has not been shown for day 3 of those infected and non-infected flies.

Thank you for bringing this to our attention. We have now added “ns” to the figure, and to the statistical analysis details in Supplementary Table S4.

However, it was claimed "No effect was seen after three days exposure (better to be "after exposure for three days") to B. bassiana…" (line 114-116). It is confusing regarding the descriptions of Figure 1C legend: 1) Not to be "3-days-old vs 7-days-old but "days after infection";

Indeed, thank you for spotting these, which have now been corrected.

p values appear twice: "p=0.0122" won't be "**p<0.01", and "p=0.0048" won't be "***(actually ****shown in figure) p<0.001".

The asterisks within the graphs (eg **p<0.01) indicate the results of the multiple comparisons corrections tests. The p values the Reviewer 1 is referring to are the Two-Way ANOVA group tests. For each Two-Way ANOVA group test, three p values result for: interaction, row factor and column factor. These p values test the significant of the whole data set, and they are distinct from the multiple comparisons corrections test that validate specific comparisons between samples. **We have added further details in Figure 1 legend page 37 lines 1089-1095 of tracked version** **to make this point more explicit.**

Lines 125 and 127 and elsewhere, change "B. bassiana-GFP were" to "B. bassiana-GFP cells were".

done

Figure 1 and Figure S1, check out the magenta-type whole brain, stained with DAPI or phalloidin?

Figure 1 has no magenta. Figure S1 it is DAPI.

Figure 2 it is DAPI, and in Figure 3 it is repoGAL4/UAS-tomato

Line 183-184, "mtk mRNA was upregulated" or NOT since "ns p=0.0634" is shown on Figure F?
now corrected, clarification added page … line…

Line 220, and sarm.
thank you, now corrected

Line 570, there is a citation issue here.
thank you, now corrected

— — —

Reviewer #2

The authors describe a role for Toll signaling in detrimental neuronal loss associated with Beauveria bassiana fungal infection in Drosophila melanogaster model. They show that neuronal loss and associated defects in climbing ability (and to some extent in survival from the infection) requires Toll signaling branch via wek/Sarm. The authors have done major work on the manuscript since the previous round of commenting via Review commons, and the conclusions are now backed up with stronger evidence. I'm happy with the manuscript as it is now, below just a few notes I'd like the authors to respond and minor suggestions.

We are grateful to this Reviewer for the positive evaluation of our revised version.

I'm still pondering the choice of controls. I would think that crossing the driver line to the genetic background of the RNAi lines used (available at the stock centers) is the best way to control for the (possible) genetic background effect. For me, the benefit of outcrossing the GAL4/UAS constructs to a "random" wild type strain seem an odd control for experiments where RNAi strains in well characterized backgrounds are used. This said, I do not doubt the validity of the results in this manuscript, considering that they have been confirmed with multiple RNAi strains and wt flies.

We value the Reviewer’s feed-back and we’ll keep this in mind for future projects.

Line 254 onwards/Figure 5 I-J. It is said in the text that Toll-1 RNAi increased survival (albeit not significantly) but did rescue the climbing phenotype. To me, this seems to be the opposite: The survival of Toll-1 RNAi flies from infection does not significantly differ from the survival of the uninfected flies of the same genotype (hence rescues it), whereas in the case of climbing, the ability is improved but remains significantly worse than in controls.

Reviewer 2 is correct in pointing out that in Figure 5I: infected *tubGAL80ts, MyD88>Toll-1-RNAi* flies have increased survival that is not statistically significantly different from non-infected controls, meaning survival is rescued. However, when compared to infected genetic controls, the survival of infected *tubGAL80ts, MyD88>Toll-1RNAi* flies is not significantly different from that of flies bearing *Toll-1RNAi* but lacking a GAL4 driver. Thus, one cannot conclude that the rescue observed was due to the knock-down on *Toll-1*. To conclude, the result is not robust compared to both non-infected and genetic controls, although it suggests that acute adult-restricted knock-down of Toll-1 improves survival.

By contrast, the result in Figure 5J shows that climbing by infected *tubGAL80ts, MyD88>Toll-1RNAi* flies is statistically significantly improved compared to that of infected genetic controls but differs from non-infected controls. This means that acute, adult specific Toll-1 knock-down improves climbing, although it does not achieve the normal climbing of non-infected flies.

In response to this Reviewer’s point, **we have improved the explanations in the text, page 10 lines 301-308 and page 14-15 lines 456-470 of tracked version.**

I find the strong increase in survival of the Toll RNAi flies rather surprising, considering that via using the MyD88>, Toll-1 is likely silenced in most fly tissues, including the fat body, the major organ producing AMPs via Toll and Imd pathways. One could think this would be detrimental in the overall humoral immune response against the fungi, but, based on the data here, clearly is not. This is something I'd like the authors to comment on (also in the manuscript text). (If after specific effects on brain function, one could have opted for a neuron-specific driver).

As explained above, even if dramatic, it is not a ‘strong increase in survival’ but rather a not-significant increase compared to genetic controls. Still, the improvement in survival and climbing is indeed remarkable, and consistent with the rescue of *B. bassiana*-induced neurodegeneration. We had already explained in the discussion of our revised version that acute, adult restricted Toll-1 RNAi knock-down was different from the commonly used method to infect Toll-1 mutant flies by pricking with an infected needed. **This is explained in page 15 lines 454-460 of this R2 version tracked version. Additional explanation has been given in lines 461-470 of tracked version.**

We had also already explained in the Discussion section that the data also suggested that *B. bassiana* induced neurodegeneration most likely involves also other Toll-1 dependent and independent mechanisms rather than just going via Wek and Sarm. In particular, we mentioned the possibility that fungal volatiles and Toll-1 induced antimicrobial peptides could also potentially drive neurodegeneration. This would also explain why knocking-down *Toll-1* acutely in the adult would rescue behavioural phenotypes, and more robustly than knocking-down *sarm***.**

**This can be found in page 16, lines 471-482 of tracked version of this revised R2 version.**

For the revision, we also carried out a trial test of survival and climbing with a neuronal driver (*tubGAL80ts, nsyb-GAL4>Toll-1RNAi*). However, the results were less robust than with *MyD88-GAL4*. This difference can be explained as follows. Firstly, *nsyb-GAL4* is expressed in all neurons, not only those expressing *Toll-1, wek* and *sarm*, but including neurons expressing *Toll-1* but not *wek* or *sarm,* and viceversa. Thus, any effect of knocking down the genes could have been diluted or compounded. Secondly, *nsyb-GAL4* is expressed only in neurons, not in glia, whereas *MyD88-GAL4* is expressed in some neurons and some glia. We had shown that *B. bassiana* caused glial cell loss via Toll-1, thus the contribution of glial cells to the phenotypes would have been lost using *nsybGAL4*-driven knock-down. In fact, the milder effect obtained with *nsybGAL4* compared to that using *MyD88Gal4* suggests that Toll signalling within glial cells, leading to glial cell loss, is an important contributing factor to *B. bassiana*-induced neurodegeneration. Finally, we explained in the Discussion that our data suggest that the Toll-1/wek/sarm mechanism is most likely not the only one inducing neurodegeneration upon fungal infection and fungal volatiles and Toll-1 induced antimicrobial peptides could contribute to neurodegeneration too. We concluded that it was best to complete the revision using MyD88-GAL4 as not only it gave more robust behavioural phenotypes, but it also made it possible to directly link the cell biology data to the behaviour data. Repeating the cell biology with *nsybGAL4* was beyond the scope of a revision, as the work had already taken almost 5 years to complete.

Line 313 Toll10b misspelled

Thank you, now corrected.

Lines 1133 and 1166 refer to Table S4, but cannot find such a Table

Supplementary Table S4 contains the statistical analysis, and it had been submitted.

FM4-64 staining looks quite similar exposed and unexposed brains. What are the bright green areas detected in both?

FM4-64 stainings of the Drosophila brain did produce background (Supplementary Figure S1A). The thready green areas are most likely tracheae, as the trachea is “sticky” and gets picked up non-specifically by multiple stainings. The other spots seem to be non-specific background. By contrast, in the exposed brains, we focused on FM4-64 staining that looked like cells and had nuclei, as they were also DAPI+, and these FM4-64+ DAPI+ cells were smaller than Drosophila brain cells (Figure 2A).

As this dye produced such background, we also used two other ways to visualise fungal cells: calco-fluor white and transgenic *B. bassiana-GFP*. Each method has strengths and weaknesses, so using three distinct methods enabled us to obtain robust data.

One could mention also in Figure 3 F&G or in the Figure caption that these are OR flies
Thank you, this has now been added to the figure legends of Figure 3F,G and also Figure 3A.

— — —

Reviewer #3

In the original submission, the authors showed fairly robust evidence that exposure to B. bassiana fungus leads to loss of brain cell function and that several host immune genes are required for this interaction. While the main conclusions were largely supported, the reviewers had some concerns relating to factors like whether the fungus was confirmed to be within the brain or not. The authors have put impressive effort into the revisions, which addresses all comments. Many new figures have been added and text has been revised as recommended by reviewers. Importantly, they have put in several new fluorescence microscopy figures that show negative controls and others that demonstrate the presence of the fungus in and around the brain, among other new additions. With all major concerns addressed, the manuscript will be a strong addition to the literature.
We would like to thank Reviewer for the positive evaluation of our revision, and we are please they are satisfied with our revised version.
