# Supplementary material for: Toll-1-dependent immune evasion induced by fungal infection leads to cell loss in the Drosophila brain
Source: PLoS Biol. 2025 Feb 13;23(2):e3003020. doi: 10.1371/journal.pbio.3003020 (PMC11825051; doi:10.1371/journal.pbio.3003020)
Supplement: Attachment — Submitted filename: 146945_1_related_ms_3100041_shnn37.docx [file pbio.3003020.s012.docx]

**Manuscript number:** **RC-2024-02514**

**Corresponding author(s):** Alicia, Hidalgo

*[The “revision plan” should delineate the revisions that authors intend to carry out in response to the points raised by the referees. It also provides the authors with the opportunity to explain their view of the paper and of the referee reports.*

*The document is important for the editors of affiliate journals when they make a first decision on the transferred manuscript. It will also be useful to readers of the reprint and help them to obtain a balanced view of the paper.*

*If you wish to submit a full revision, please use our "*[*Full Revision*](https://reviewcommons.org/templates/full-revision.docx)*" template.* ***It is important to use the appropriate template to clearly inform the editors of your intentions.****]*

## 1. General Statements [optional]

*This section is optional. Insert here any general statements you wish to make about the goal of the study or about the reviews.*

Please find uploaded a revision in progress, ie a **preliminary and unfinished revision**.

Most importantly, we provide new evidence:

**1. *B. bassiana* penetrates the brain: in** **Revised Figure 2C-F**, we show *B. bassiana-GFP* in the location where the proboscis enters the brain (**Figure 2C**), crossing the BBB (**Figure 2F**) and located deep into the brain (**Figure 2D**). We also provide **higher magnification images of the abundant germinating spores** **detected in the brain (Figure 2).**

2. **MyD88>hisYFP is expressed in glial cells that line up the brain and contributes to the BBB: Supplementary Figure 2.** As *B. bassiana* can induce Toll-1-signalling dependent glial cell death, in this way *B. bassiana* could make holes in the BBB making its way into the brain.

3. Non-infected fluorescent microscopy images controls (**Supplementary Figure 1)**.

Our **planned revisions** to address Reviewers’ comments and following their recommendations, involving new experiments are in brief:

1. To test whether feeding flies with *B. bassiana-GFP,* without any further exposure, is sufficient for *B. bassiana-GFP* to penetrate the brain. We will combine it with a membrane glial marker to look for partial damage of the BBB surrounding the proboscis/esophagus as it traverses the brain.

2. To test whether knock-down of Toll/wek/sarm rescues the climbing impairment of infection and test whether their over-expression is sufficient to impair climbing in the absence of infection.

As these experiments require lengthy genetics, we will be able to produce results by October but not sooner.

3. We will build and provide a statistical analysis file.

4. We will add the data dots onto the requested graphs.

We have already carried out most corrections to manuscript text that were possible at this stage. Please find uploaded the preliminary revision – ahead of the deadline.

## 2. Description of the planned revisions

*Insert here a point-by-point reply that explains what revisions, additional experimentations and analyses are planned to address the points raised by the referees.*

| **PLANNED REVISIONS: EXPERIMENTS AND OTHER DEMANDING TASKS** | | | |
| --- | --- | --- | --- |
| **REVIEWER** | **CRITICISM** | **EXPERIMENT** | **WHEN BY** |
| **Reviewer 1**  **Reviewer 1** | **1. ….. fungal spores can barely survive the highly-alkaline foreguts. It is questionable that flies could ingest spores and spores "infiltrated" the brains.** | ***B. bassiana-GFP* TEST:**  **Feed flies with *B. bassiana-GFP* and test 1, 2, 3 and 7 days later see if GFP found inside the proboscis and brain.**  **Feeding, dissect brain and proboscis, stain with anti-GFP, confocal microscopy 488nm.**  **Scan proboscis and brain.** | August |
|  |  | **We also provide images showing B. bassiana-GFP at the point of entry of the proboscis into the brain: revised Figure 2F** | **Added new data** |
|  | **2. detection of fungal SPORES in fly brain THREE days post exposure (page 5) by infiltration. It would be impossible that, even fungus could breach the blood brain barrier (BBB), it might be the fungal hyphae/mycelia but not the spores.** | **We have** now added higher magnification **images** of *B. bassiana-GFP* in brain, crossing the BBB and of germinating spores: **see revised Figure 2C-F.** | **Added new data** |
|  | **3. … authors provided the evidence of the damaged BBB SEVEN days post exposure, a few days LATER than the detection of fungal spores in brains (THREE days) post treatment mentioned above. Did "spore infiltration" occur before BBB damage?** | **Test B. bassiana-GFP in repo>tomato flies:**  **Feed flies with bassiana-GFP and test whether there is a correlation between GFP+ and damage to BBB, using repoGAL4/UASTomato.**  **Test at 1, 3 and 7 days post-ingestion.**  **Focus on area where proboscis enters brain, and scan at 63x to see if holes in BBB can be detected** | August |
|  | **by day seven more than half of the flies had died" (Fig. 1B). It is questionable therefore that the "other half" of the diseased and dying insects were used for the following experiments….. The brain function of dying animals could be compromised by multiple direct or indirect factors.** | **Add videos** showing infected flies look healthy at 7 days post-infection | August |
| **Reviewer 2** | **1. As the initial experiments (including the effect on survival and climbing assay) have been performed using OR/CantonS, it would be interesting to investigate if the same is seen with a more similar background to that what is used in the genetic experiments.** | **CLIMBING ASSAY**  Add control:  MyD88GAL4; tubGAL80ts x oregon  Sarm GAL4[NP0257] x oregon | October |
|  | **2. To see if the Toll (or wek/sarm) RNAi in the brain rescues the climbing defect caused by the fungal infection.** | **CLIMBING ASSAY**  **Test rescue of fungi-dependent climbing impairment by knocking-down Toll/wek/sarm in the brain**  **Test 1 in** **infected and not-infected** flies, with standard *B. bassiana,* 7 days:  **MyD88GAL4; tubGAL80ts x**  UAS-Toll-1RNAi[KK]  UAS-wekRNAi  UAS-sarmRNAi  Controls:  MyD88GAL4; tubGAL80ts x  Oregon  UAS-Toll-1RNAi[KK] x oregon  UAS-wekRNAi x oregon  UAS-sarmRNAi x oregon | October  flies need to be bred first in large numbers (4 weeks), crosses set up, F1 bred at 18°C for 3 weeks, then flies are exposed to B. bassiana and then experiments done, in 3 biological replicated.  **This requires a minimum of 8 weeks.** |
|  |  | **Test 2 in** **infected and not-infected** flies, with standard *B. bassiana* 7 days:  **tubGAL80ts; nsybGAL4 x**  UAS-Toll-1RNAi[KK]  UAS-wekRNAi  UAS-sarmRNAi  Controls:  tubGAL80ts; nsybGAL4 x  Oregon  UAS-Toll-1RNAi[KK] x oregon  UAS-wekRNAi x oregon  UAS-sarmRNAi x oregon | October |
|  |  | **Additional experiment:**  Test whether over-expression of *Toll-1[10b], wek* or *sarm* impair climbing in the absence of infection:  **tubGAL80ts; nsybGAL4 x**  **UAS-Toll-1[10b]**  **UAS-wek**  **UAS-dsarm** | September |
| **Reviewer 3** | would be helpful to **know if earlier time points were ever checked for BBB damage, loss of brain cells, or presence of fungus** etc. | We did try earlier time points for some experiments, not for all.  **To discuss.** |  |
|  | • Figures 2A, 2D, 2E, 3E, & 3H: If multiple replicates or samples are represented in the data, it would help to be able to see the data points underlying these bars. If so, please add them to the graphs to see the spread of data points. | **Change graphs’ style.** |  |
|  | • It would be ideal if data underlying data points and full statistical models and outputs could be included through a public repository such as Dryad. This would be ideal for full assessment of statistical approaches | We have not used **Dryad** before, but willing to learn.  At the very least we will provide an **excel table with all the statistical details.** |  |

## 3. Description of the revisions that have already been incorporated in the transferred manuscript

*Please insert a point-by-point reply describing the revisions that were already carried out and included in the transferred manuscript. If no revisions have been carried out yet, please leave this section empty.*

| **Changes already made** | | |
| --- | --- | --- |
| **Reviewer** | **Comment** | **Revision: where** |
| **Reviewer 1** | However, it was unclear how those flies could be uniformly contaminated with fungal spores in the infection chamber shown in Fig. 1A, by landing on fungal "carpet"? | To clarify this, we have now added further details on the experimental set-up in the manuscript text, page 5 lines 132-134 and in Materials and Methods section, page 18, lines 528-533 |
|  | Fungal spores can be ingested by insects, largely those with chewing mouthparts. However, fungal spores can barely survive the highly-alkaline foreguts. It is questionable that flies could ingest spores and spores "infiltrated" the brains. | **Added citation of Ishii et al (2017, Scientific Reports),** who demonstrated that *B. bassiana* causes mosquito death only when ingested through the proboscis. This is consistent with our feeding assay data, which demonstrate that adult flies did ingest spores (Fig 2A). |
|  |  | Similarly to other reports that did not qualify whether they detected spores or hyphae(Elya et al 2018 eLife; Ishii et al 2017 Scientific Reports), **we have now edited the term “spores” for a generic “fungal cells” for the result using FM4-64 and calcofluor white, see page 5 line 143.** We have kept the term spores for the results obtained with *B. bassiana-GFP*, as we saw also germlings or germinating spores. We have made this explicit in **page 6 line 150.** |
|  |  | **We have also included additional, higher magnification images showing germinating spores (also called germlings) in the brains of exposed flies (revised Figure 2E).** |
|  |  | We have added text on these points in: page 6 lines 153-157 and Discussion page 12 lines 363-368 to Page 13 line 384-404. |
|  | Regarding the detection of fungal cells in fly brains, on the one hand, the authors argued that detection of fungal SPORES in fly brain THREE days post exposure (page 5) by infiltration. It would be impossible that, even fungus could breach the blood brain barrier (BBB), it might be the fungal hyphae/mycelia but not the spores. One the other hand, the authors provided the evidence of the damaged BBB SEVEN days post exposure, a few days LATER than the detection of fungal spores in brains (THREE days) post treatment mentioned above. Did "spore infiltration" (even impossible) occur before BBB damage? | **To clarify this point, we have added a comment in page 8 line 230-231: “**Importantly, as glial cells form the BBB in Drosophila, loss of glial cells could also facilitate entry of *B. bassiana* into the brain”. |
|  |  | **We also now provide evidence** that MyD88>hisYFP is expressed in glial cells **(Supplementary Figure 2A,B,D)** and contributes to the BBB **(Supplementary Figure 2C).** As we showed that B. bassiana caused Toll-1dependent glial cells loss, this means that B. bassiana could damage the BBB by inducing glial cell death and thus holes in the BBB. A comment has been added to **page 9 lines 276-286.** |
|  | The authors stated that "by day seven more than half of the flies had died" (Fig. 1B). It is questionable therefore that the "other half" of the diseased and dying insects were used for the following experiments. There would be no wonder that the climbing of these diseased and dying flies was impaired, however, which could be due to muscle damage, hemocyte number decline and reduction of energy production etc. apart from brain cell loss. The brain function of dying animals could be compromised by multiple direct or indirect factors. | Flies that were immobile, dying or stuck to the fungal culture were not used. Selected flies were inserted into fresh vials and flipped twice, to remove excess spores from their cuticles.  **This has been clarified in page 18 lines 530-535.** |
| **Reviewer 2**  **Reviewer 2** | It is somewhat unclear what are the controls in the genetic experiments. For example, in Figure 2, the control is UAS-TrpA1/+. Does this mean that the UAS-TrpA1 flies have been crossed to something (like the driver background strain) or used as it is? In Figure 4, controls are ">+". Again, are MyD88>histoneYFP;tubulinGal80ts flies crossed to something (in this case, maybe the w1118 background of the KK library RNAi strains) or used as homozygous? And same for the subsequent figures. I'd ask the authors to clarify these points in the manuscript. | We have clarified all the genotypes in the manuscript, as requested: see in **Materials and Methods page; figure legends for each figure; and we will provide an excel file that contains all statistical analysis details and includes the full genotypes (Table Sx TO DO).** |
|  | Could the authors please explain why they opted for MyD88-GAL4 in the experiments in Figures 5-7? | **We have now added a clarification** on this point in **page 8 lines 244-245 and page 9 lines 251-252.** |
|  | What is the overall expression pattern of MyD88-GAL4? | We published this in Li et al (2020) eLife. We have added a comment in **page 9 lines 252-253.**  We **now provide further data** showing that MyD88>hisYFP is expressed in glial cells that line up the edge of the brain **(Supplementary Figure 2A,B,D)** and contributes to the BBB **(Supplementary Figure 2C).**  As the BBB is formed of surface glia in *Drosophila,* and we had already shown that B. bassiana exposure causes glial cell loss, this could explain how *B. bassiana* makes its way into the brain. |
|  | How do the flies survive the infection with Toll knockdown in MyD88-epressing cells (expressed at least in all immunogenic tissues)? A bit more explanation would clarify the situation. | **We have now added a comment** in the Discussion session, **page 15 lines 445-450.** |
|  | Page 3: Full species name should be given here (Drosophila melanogaster) | We have now corrected this. |
|  | A short description of the FM4-64 dye (what it stains etc) would be useful for the readers unfamiliar with it. | We have now added this to the Materials and Methods section**, page 18 lines 538-544.** |
|  | Page 6: Please explain shortly why TrpA1 overexpression was used to activate the neurons | We have added an explanation in Materials and Methods **in page 20 lines 595-598** |
|  | Figure 2E: What is the genotype of the flies? mtk is lacking statistics | The genotype is wild-type (Oregon), and this information has been added. |
|  | Page 8: third row refers to Figure 2 but should be Figure 3. | We have now corrected this. |
|  | Although antibody stainings are performed using "standard methods", a short overview on the process should be presented also in the current manuscript. Also, I imagine fungal spores are all over the flies retrieved from the infection chamber. I'd like to know (and this could also be described in the materials) how the flies (and the brains) were treated/washed prior to preparing brains for immunostaining and imaging? | This has now been added to the Methods on the antibody stainings. **page 22 line 676-677, page 23 lines 678-689**  On the second point, to prepare flies for immunostaining, infected flies were flipped twice in vials to remove excess spores. They were then wash in ethanol followed by a 10-minute wash in PBS before dissecting the brains. This has also been added to the Methods section. |
|  | Some typos and inconsistencies at various places. For example, at some occasions B. bassiana written without a space in between "B" and "bassiana" and not in italics (both in figures and in text); on page 5, first line: "mimicked" misspelled | we have now corrected them. |
|  |  |  |
| **Reviewer 3** | negative controls of fluorescence microscopy imaging were not always included | Added now as new Supplementary Figure 1. |
|  | Also, for all positive samples, was presence of the fungus noted in all samples? | Numbers added to revised Figure 2 legend. |
|  | • It would help to have line numbers throughout | We have now added line numbers to manuscript. |
|  | • Figure 1- what are the arrows in panels D-G? | **We have now added the arrows into the figure legends,** as they were missing. |
|  | • Methods: A few details are unclear: o Was only one fly sex used or were both used for the various assays? If both were used, were they statistically assessed for differences? Sex is only mentioned in a couple of the methods sections. | **We have now included this information in the Methods section,** **page 23 lines 686-689.** |
|  | How old were the flies at the start of the experiments? A few experiments noted age, but it was not clear for all | We have now included this information in the Methods section, **page 18 line 531.** |
|  | For longevity, was the fungal culture ever replaced during the experiment? | **We have now added this information in page 18, lines 534-535.** |
|  | For the climbing assay when the flies were initially flipped, how much time was there between flips? | **A clarification has been added** |
|  | Figure 3F- what do arrows indicate? | **We have now clarified this in all the figure legends.** |
|  | • It is interesting that Wek-RNAi with infection not only rescues loss caused by the infection alone, but also increases YFP cells beyond the uninfected controls (Figure 5C). The same is true with toll-1 RNAi (Figure 4C). Why might this be? | Both Toll-1 and wek have been shown to be required to induce apoptosis (Foldi et al 2017 JCB). Thus, it is possible that knocking them down enables cells that would normally die, to survive.  **We have added a comment in Discussion, page 14 line 426-428.** |
|  | Check italicizing throughout- missed a few "Drosophila" or "B. bassiana" in main text or figures | We have now done this. |
|  | Looks like no space between C. and elegans in C. elegans in a few cases | We have now corrected this. |
|  | • Word missing: "No effect was seen after three days exposure to B. bassiana, but seven days exposure impaired climbing"... seven days of exposure? | we have now corrected this |
|  | Toll-1 misspelled pg 6 last paragraph | we have now corrected this |

## 4. Description of analyses that authors prefer not to carry out

*Please include a point-by-point response explaining why some of the requested data or additional analyses might not be necessary or cannot be provided within the scope of a revision. This can be due to time or resource limitations or in case of disagreement about the necessity of such additional data given the scope of the study. Please leave empty if not applicable.*

| **Not necessary or beyond scope of a revision** | | |
| --- | --- | --- |
| **Reviewer** | **Criticism** | **Response** |
| **Reviewer 1** | The authors stated that "by day seven more than half of the flies had died" (Fig. 1B). It is questionable therefore that the "other half" of the diseased and dying insects were used for the following experiments. There would be no wonder that the climbing of these diseased and dying flies was impaired, however, which could be due to muscle damage, hemocyte number decline and reduction of energy production etc. apart from brain cell loss. The brain function of dying animals could be compromised by multiple direct or indirect factors. | **…. after seven days of exposure to the fungal spores, only active flies were selected for experiments.** Flies that were immobile, dying or stuck to the fungal culture were not used…..  This has been clarified. |
|  | Issue of Fig. 2D labelling. | What is the issue? we cannot see anything unusual. |
| **Reviewer 2** | Is there a possibility that some of the effects seen could arise from the Toll/sarm/wek knockdown elsewhere in the fly? | Rescues within the brain observed with cell biology (eg His-YFP, antibodies) are highly unlikely to result known RNAi knock-down outside the brain, in other organs or tissues. |
|  | I wonder if, similarly to mammalian macrophages, hemocytes could be responsible for delivering the fungal cells into the brain? | There is evidence that macrophages can cross the BBB in Drosophila (Winkler et al 2021 Sci Adv). However, investigating this is a large task, that is beyond the scope of this revision. Furthermore, as the BBB in Drosophila is made up of glial cells, and we showed that exposure to B. bassiana causes glial cell loss (ie Repo+ cells), then Toll-1 dependent loss of glial cells at the BBB is a likely route to causing BBB damage.  We have now provided a new figure showing expression of MyD88>hisYFP in Repo+ glia that line up the brain **(new Supplementary Figure 2A)** and that it contributes to the **BBB (new Supplementary Figure 2C).** |
| **Reviewer 3** | that the control fly genotypes used in experiments were not always the most ideal controls (eg, compared WT genotypes to RNAi against a gene of interest; ideally would be RNAi against a control gene compared to RNAi against a gene of interest), | Although this would have been a good control for RNAi, it is not common practice, as most publications using RNAi do not carry this out. Unfortunately, we are not able to mend this at this stage. The manuscript is the result of 4 years of very hard work. Each experimental block was carried out with all genotypes treated at the same time, together. This is the best way to make data comparable across genotypes and infected vs not-infected. We would not be able to repeat everything now in order to incorporate such a control, as this would take us another 4 years.  **Reviewer 3 recommended to discuss this a caveat, which we have done in the discussion, page 14 lines 422-425..** |
|  | Other responses or experiments may also be appropriate to address some of the major concerns- maybe additional assay(s) of brain function other than climbing? | Thank you for the suggestion. However, tests for brain function are not trivial and we consider this beyond the scope of this manuscript. Furthermore, we do not see how another behavioural test would add information that the current data have not already provided. |
